# Shotgun metagenomics reveals the flexibility and diversity of Arctic marine microbiomes

**DOI:** 10.1093/ismeco/ycaf007

**Published:** 2025-01-21

**Authors:** Nastasia J Freyria, Thais C de Oliveira, Arnaud Meng, Eric Pelletier, Connie Lovejoy

**Affiliations:** Department of Natural Resource Sciences, McGill University, Ste. Anne-de-Bellevue, Québec, QC H9X 3V9, Canada; Département de biologie, Québec Océan, Université Laval, Québec, QC G1V 0A6, Canada; Institut de Biologie Intégrative et des Systèmes Université Laval, Québec, QC G1V 0A6, Canada; Institut de Biologie Intégrative et des Systèmes Université Laval, Québec, QC G1V 0A6, Canada; Centre d’Étude de la Forêt, Faculté de Foresterie, de Géographie et de Génomique, Université Laval, Québec, QC G1V 0A6, Canada; Institut Pasteur, Université Paris Cité, Metabolomics Core Facility, Paris, 75015, France; Génomique Métabolique, Genoscope, Institut de Biologie François Jacob, CEA, CNRS, Université Paris-Saclay, Evry, 91000, France; Génomique Métabolique, Genoscope, Institut de Biologie François Jacob, CEA, CNRS, Université Paris-Saclay, Evry, 91000, France; Research Federation for the Study of Global Ocean Systems Ecology and Evolution, FR2022/Tara GOSEE, Paris, 75000, France; Département de biologie, Québec Océan, Université Laval, Québec, QC G1V 0A6, Canada; Institut de Biologie Intégrative et des Systèmes Université Laval, Québec, QC G1V 0A6, Canada

**Keywords:** arctic phytoplankton, polar microbiome, functional metagenomics, metagenome-assembled genomes (mag), 18 s rRNA gene, arctic ecology, Pikialasorsuaq

## Abstract

Polar oceanographic regions are exposed to rapid changes in temperature, salinity, and light fields that determine microbial species distributions, but resilience to an increasingly unstable climate is unknown. To unravel microbial genomic potential of the Northern Baffin Bay’s polynya, we constructed eight metagenomes from the same latitude but targeting two sides of *Pikialasorsuaq* (The North Water) that differ by current systems, stratification, and temperature regimes. Samples from the surface and subsurface chlorophyll maximum (SCM) of both sides were collected 13 months apart. Details of metabolic pathways were determined for 18 bacteria and 10 microbial eukaryote metagenome-assembled genomes (MAGs). The microbial eukaryotic MAGs were associated with the dominant green algae in the Mamiellales and diatoms in the Mediophyceae, which tended to respectively dominate the eastern and western sides of *Pikialasorsuaq*. We show that microbial community taxonomic and functional signatures were ca. 80% similar at the latitude sampled with only 20% of genes associated with local conditions. From the metagenomes we found genes involved in osmotic regulation, antifreeze proteins, and photosystem protection, with hydrocarbon biodegradation and methane oxidation potential detected. The shared genomic compliment was consistent with adaptation to the Arctic’s extreme fluctuating conditions, with implications for their evolutionary history and the long-term survival of a pan-arctic microbiome. In particular, previously unrecognized genetic capabilities for methane bio-attenuation and hydrocarbon metabolism in eukaryotic phytoplankton suggest adaptation to dark conditions that will remain, despite climate warming, in the high latitude offshore waters of a future Arctic.

## Introduction

Arctic summer sea-ice extent and thickness have declined by over 30% over the last three decades [1] and the consequences of a longer ice-free season will vary by region [2–4]. Marine food webs are predominantly underpinned by marine microbes: microbial eukaryotes (phytoplankton and other protists), bacteria, archaea and viruses. In contrast to tropical and temperate oceans, in the Arctic Ocean, eukaryotic phytoplankton dominate the photosynthetic functional compartment [5] with picophytoplankton (0.2–3 *μ*m), in particular, *Micromonas polaris* (Chlorophyta) being ubiquitous. Arctic nanophytoplankton (2–20 *μ*m), are more diverse and include *Phaeocystis pouchetii* (Haptophyta), and small *Chaetorceros* spp. (Bacillariophytina) [5–7]. Heterotrophic protists and bacterioplankton (Bacteria) in the upper water column recycle organic matter as part of the microbial food web [8, 9]. In the Arctic, protists are characteristic of water masses [10] and bacterioplankton include species uniquely adapted to Arctic conditions and available substrates [11, 12]. Viruses and Archaea have a role in remineralization of nutrients and are implicated in other processes controlling biogeochemical cycles [9]. Archaea have been associated with the different Arctic water masses defined by temperature, salinity and nutrient ratios [13, 14] suggesting their use as water mass indicators. Viruses likely follow their hosts [15] and may also be indicative of water masses.

Surface salinity controls upper water column stratification and along with temperature is associated with species distribution and diversity of microbial communities [10]. High throughput amplicon sequencing in multiple Arctic regions, including offshore in the Canada Basin [16], marine fjords of Svalbard [17], and Hudson Bay [18] highlight the diversity of both bacteria and microbial eukaryotes. The Tara Ocean Arctic Circle reported on the metagenomic specialization of Arctic microbes [19, 20].

The longer naturally occurring ice-free season the North Water region of Northern Baffin Bay, referred to by its Inuit-Greenlandic name *Pikialasorsuaq* [21, 22] can be thought of as an analog for a future more open summertime Arctic Ocean, with the two sides representing two future scenarios [23]. One under the influence of increased freshening and the other with Atlantic water penetration into the Arctic. Specifically, the Canadian side (W°) is influenced by a strong southward flow of cold nitrate and silica rich water [24], while the Greenland side (E°) is strongly influenced by Atlantic water from the warmer north flowing West Greenland Current, which upwells along the coast of Greenland [25]. Earlier microscopy studies reported characteristic phytoplankton communities [26] at sites on the two sides of *Pikialasorsuaq* [6] and more recently high-throughput amplicon sequencing found a clear separation of the east and west side communities in August [23]. A multiyear study of the <3 micron size fraction carried out in the same region indicated a strong seasonal influence on the communities [5]. However, to our knowledge, this is the first metagenomic analysis of microbial functional pathways of the two sides.

**Figure 1:**
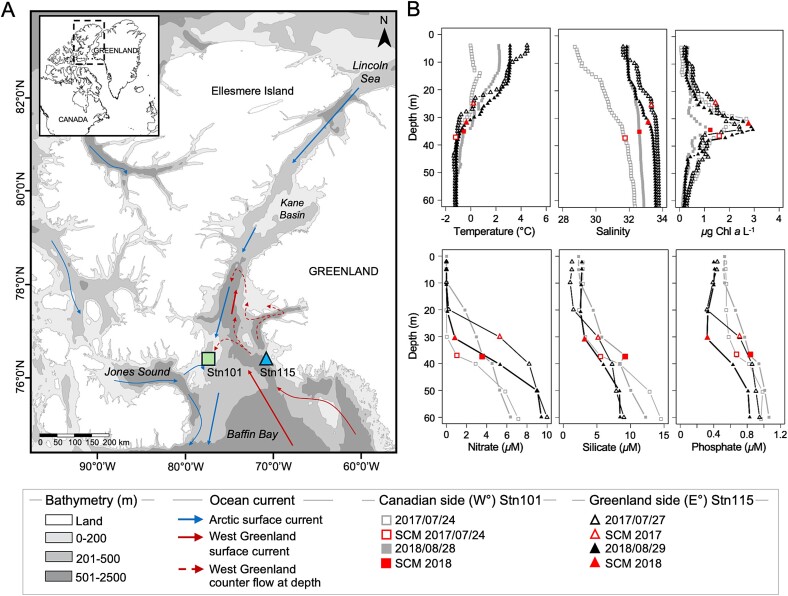
**Map of the sampling region and vertical profiles of environmental parameters.** (**A**) Map of the two sampling stations showing the circulation within the *Pikialasorsuaq* region adapted from Melling et al.*,* [25]. The map was generated using ocean data view (v.4.7.8, http://odv.awi.de). (**B**) Vertical profiles of the water column from surface to 60 m deep of temperature, salinity, chlorophyll a fluorescence, and nitrate, silicate and phosphate concentration. Acronyms: subsurface of chlorophyl maximum (SCM).

We hypothesized that lower salinity in the surface waters on the colder more ice-influenced Canadian side would have more euryhaline species and be adapted to salinity stress [27, 28], and an analog to regions strongly affected by multi-year and glacier ice melt, which leads to surface freshening. Whereas the slightly warmer waters on the Greenland side would show higher genetic potential for defence metabolites associated with higher grazing and parasitism in the more productive waters. Due to the ongoing productivity that occurs in the region in summer [29], we expected both microbial eukaryotes and bacterial communities would have a large repertoire of organic carbon degrading pathways, that would facilitate nutrient regeneration in the upper waters where they remain into autumn [30]. Here, we determined community functional diversity using shotgun metagenome and used protist 18S rRNA amplicons to verify major eukaryotic taxa. The samples from July 2017 and August 2018, were from a range of different salinity, stratification and temperature conditions. Specifically they were from the surface (surf), and from the subsurface chlorophyll maximum (SCM), also referred to as a deep chlorophyll maximum [31], which occurs near the upper halocline across much of the Arctic Ocean. To minimize the effect of seasonality within years [5], which impacted the interpretability of for example, the Tara Ocean Survey [19], we selected two stations along the same latitude that could be sampled within 48 hours of each other, on the Canadian and Greenland sides of *Pikialasorsuaq* (Fig. 1).

## Materials and methods

Sampling and sample treatment were based on protocols provided in Freyria et al.*,* [5], additional details can be found in Supplementary Methods.

### Field sampling

Sampling took place in the context of annual multi-disciplinary ArcticNet missions on the *CCGS Amundsen* in late July 2017 and late August 2018, between Ellesmere Island, Canada, and the west coast of Greenland (Fig. 1A). To compare the two sides of *Pikialasorsuaq*, we selected stations (Stn 101 and Stn 115) at the ends of a standard transect from −71.18°W to −77.40°W longitude and 76.33°N to 76.38°N latitude [5] (Table S1). Samples were collected from surface (0–5 m) and SCM (25–37 m depths; Table S2). Seawater samples for nutrients, flow cytometry and nucleic acids were collected using a Rosette system with 12-L Niskin-type bottles and sensors as described earlier [32]. For nitrate, phosphate, and silicate measurements, water samples were filtered through 0.2 *μ*m polycarbonate (PC) filters into acid-rinsed 15 ml Falcon tubes and analyzed on board with a Bran-Luebbe 3 auto-analyzer. Nucleic acid samples were collected by prefiltering 6–8 L of seawater through 47 mm 3 *μ*m pore size PC filter, with material collected in 0.2 *μ*m Sterivex (Millipore Sigma Canada Ltd.) units. RNAlater was added to the Sterivex units and stored at −80°C until analysis.

### Nucleic acid extraction and sample preparation

DNA and RNA were extracted from the same Sterivex unit using the All-Prep DNA/RNA Minikit (Qiagen) [33]. RNA contamination from DNA samples was checked by gel electrophoresis, when DNA were found, DNAse treatment was used with RNAse-free DNAse set (Qiagen). Microbial eukaryotic community composition was determined by high-throughput 18S rRNA gene and rRNA sequencing [5]. RNA was converted to cDNA and the V4 region of 18S rRNA was amplified in both DNA and cDNA samples using eukaryotic-specific primers (E572F/E1009R) with MiSeq adaptors, followed by nested PCR [34]. PCR products were purified and quantified, with DNA quality verified using a NanoDrop 8000 spectrophotometer and a Qubit fluorometer with the dsDNA BR Assay Kit.

### Illumina MiSeq and Solexa sequencing and analyses

Amplicon libraries were sequenced at the IBIS Illumina MiSeq genomic analysis platform (Université Laval, Québec, Canada). Sequence reads were processed as described previously [5], with quality checks, filtering, de-replication, and chimeras removed (see Supplementary Methods). Operational Taxonomy Units (OTUs) were picked at >98% similarity. Taxonomic affiliation was determined using Silva v.132 [35] and PR^2^ v.4.1 [36] portals.

Metagenomic DNA was sent for library preparation at the Laboratoire de séquençage, Genoscope (Évry, France), and sequenced using an Illumina Solexa Genetic Analyzer. Sequence data were quality filtered, trimmed and assembled as detailed in the Supplementary Methods. Unassembled reads that passed quality filtering were mapped back to the assembled contigs. These unmapped reads were concatenated with the assembled contigs and uploaded onto the JGI IMG/M analysis pipeline [37], which was used for gene calling, functional annotation, and taxonomic affiliation. All metagenomes were rarefied and normalized for comparison, with gene counts adjusted to hits per million based on total reads.

### Binning of metagenomes

Details of Metagenomic binning for each of the eight metagenome assemblies to reconstruct metagenome-assembled genomes (MAGs) are given in the Supplementary methods. Briefly, eukaryotic and prokaryotic contigs were separated using EukRep v0.6.7. Multiple binning tools were employed to increase the number of bins, retaining only contigs longer than 2000 bp. The completeness and contamination of bacterial bins were assessed with CheckM v1.1.3, retaining those with less than 15% contamination and over 50% completeness. Eukaryotic bin quality was verified with EukCC v2.1.0 and bin replication was checked using dRep v3.2.2. Taxonomic classification for bacterial bins was performed with the Genome Taxonomy Database Toolkit, and functional annotation was completed using MetaErg. Analyses were conducted using Compute Canada (Alliance) facilities and in-house computers.

### Statistical analyses

All statistical analyses were conducted in R Studio. Spearman’s rank-order correlation was performed and visualized using functions from the “*Hmisc*” and “*corrgram*” packages. Constrained Correspondence Analysis (CCA) was computed to differentiate sampling stations based on geographic location and environmental variables. Independent parameters explaining variability in the CCA were selected using functions from the “*vegan*” package through automatic forward selection for optimal model building. Unweighted Pair Group Method with Arithmetic Mean (UPGMA) was applied to rarefied and normalized metagenomes for each community group (Bacteria, Eukaryota, Archaea, and virus) based on Bray-Curtis distance matrices. Non-Parametric Multivariate Analysis of Variance (NPMANOVA) tested compositional differences between the two sides of *Pikialasorsuaq* and depths (see Supplementary Method).

## Results

### Environmental setting and microbiome structure

Pelagic seawater surface and SCM samples were collected from both sides of *Pikialasorsuaq* in July 2017 and August 2018 (Fig. 1A, Table S1). Surface temperatures were cooler in 2017, −0.04°C on the Canadian side (W°) to 4.45°C on the Greenland side (E°), while 2018 was warmer (W°: 2.21°C; E°: 3.11°C; Fig. 1B, Table S1). Seawater salinity ranged from 28.72 (W°, surface 2017) to 33.57 (E°, SCM 2017). Chlorophyll *a* fluorescence was greatest on the E° side SCM in 2018. While surface nitrate was below detection limits, concentrations reached 5.32 *μ*M on the E° side in 2017 (Fig. 1B, Table S2). Silicate concentration was highest on the W° side SCM (9.12 *μ*M, 2018), and phosphate showed slightly higher concentrations on the W° side SCM (0.83 *μ*M, 2018) compared to the E° side SCM (0.71 *μ*M, 2018).

**Figure 2:**
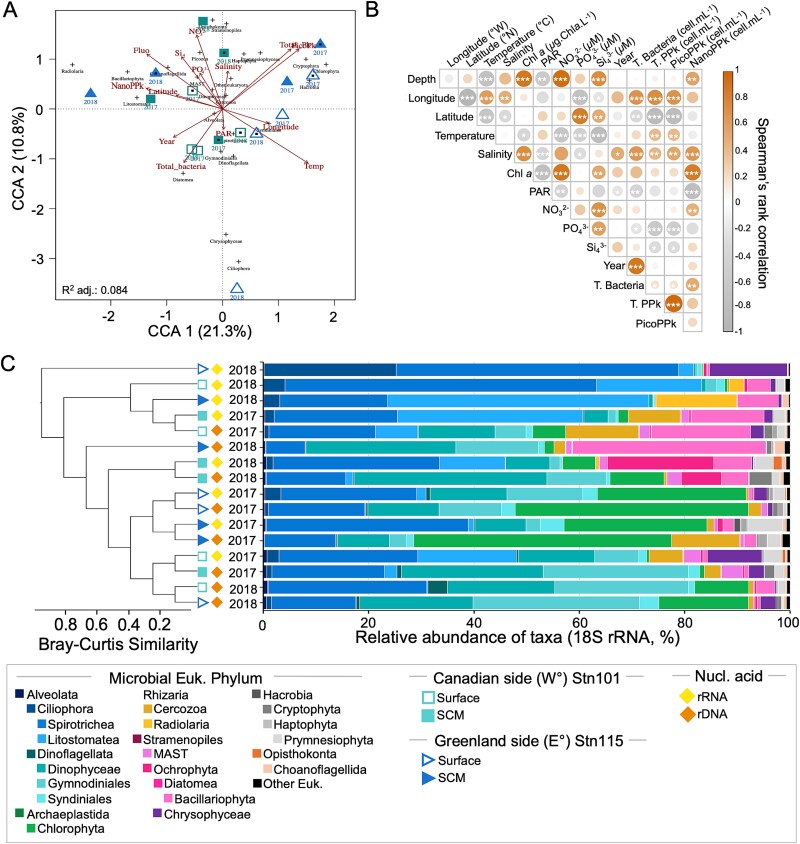
**Microbial eukaryotic community composition and clusters.** (**A**) Constrained correspondence analysis (CCA) ordination of all samples with the bray–Curtis dissimilarity measure. The 18S rRNA gene (rDNA) and rRNA reads are analysed independently, with rDNA communities indicated by a black point in the center of the symbols; rRNA lack a central point. (**B**) Spearman’s rank correlation (*r*) matrix between environmental parameters from all samples. Spearman correlation coefficients are indicated by size (the larger the circle the greater the value of the coefficients) also indicated by color shades with orange positive and greys negative. Asterisks indicate the significance level p-value from Spearman’s rho. (**C**) Relative abundance of the most abundant taxonomic groups from 18S rRNA gene (rDNA) and rRNA in samples of both years (2017–2018) for each station, and side, and both depths: surface and subsurface of chlorophyl maximum (SCM). UPGMA tree was constructed using bray Curtis dissimilarity measure. Acronyms: Depth of sampling (depth), temperature (temp.), nitrate (NO_3_^2−^), silicate (SiO^4^), phosphate (PO_4_^3−^), Chl *a* fluorescence (Fluo), downwelling irradiance (photosynthetically available radiation – PAR), total concentration of phytoplankton (T. PPk), concentration of only picophytoplankton (PicoPPk) and concentration of only nanophytoplankton (NanoPPk).

Constrained correspondence analysis revealed the relationships between environmental parameters and 18S rRNA-defined communities (Fig. 2A). The first axis accounted for 21.3% of the total variation in community structure. To assess the relationships between environmental variables and community patterns, we used the *envift()* function (*Vegan* package), which fits environmental vectors onto the ordination space. This analysis revealed several significant correlations between environmental variables and community structure. Temperature showed the strongest correlation, explaining 63% of the variation in community structure (*R^2^* = 0.63, *p*-value <0.01), followed by picophytoplankton abundance (cells <3 *μ*m) explaining 60% (*R^2^* = 0.60, *p*-value <0.01). Chlorophyll a fluorescence and total phytoplankton also showed strong correlations, explaining 55% and 53% of the variation respectively (*R^2^* = 0.55 and 0.53, both *p*-value <0.01). Nitrate concentrations explained 44% of the community variation (*R^2^* = 0.44, *p*-value <0.05). Spearman’s rank correlation revealed significant correlations between environmental variables and cell concentrations (Fig. 2B). Bacterial abundance positively correlated with salinity and negatively with phosphate. Picophytoplankton abundance correlated positively with temperature and salinity but inversely with phosphate. The highest picophytoplankton concentrations were on the E° side in 2017 (surface: 1.47 × 10^5^ cells ml^−1^, SCM: 3.33 × 10^5^ cells ml^−1^; Fig. S1A, Table S3). Nanophytoplankton concentrations correlated positively with salinity, depth, Chlorophyll *a* fluorescence and nitrate; and were lower (3.39 × 10^3^ cells ml^−1^) on the W° side surface in 2017 compared to the SCM (2.55 × 10^4^ cells ml^−1^; Fig. S1B). Bacterioplankton concentrations were 7 to 8 times greater in 2018 than in 2017 on both sides (Fig. S1C).

### Microbiome composition and distribution

After removing metazoan and fungal reads from the 18S rRNA V4 amplicon data, 181 984 reads were retained, and resulted in 5433 OTUs (4325 from 18S rRNA gene and 3694 from rRNA). Rarefaction to the sample with the fewest reads resulted in 11 374 reads per sample. Alveolata (dinoflagellates and ciliates) dominated both 18S rRNA gene and rRNA datasets (Fig. 2C). In 2017, chlorophytes, primarily *Micromonas* sp., were more abundant on the E° side, while bacillariophytes (diatoms), predominantly *Chaetoceros* sp., were more common on the W° side (Table S4). Among Rhizaria, Cercozoa were more abundant in 2017, while Radiolaria prevailed in 2018 on both sides. Community composition differed significantly between sides and depths (NPMANOVA, *n* = 16, *F* = 4.03, *p*-value <0.01). UPGMA clustering showed similarities between surface and SCM samples from the W° side in 2017 based on 18S rRNA gene and rRNA reads.

Shotgun metagenomes captured a fuller spectrum of the dominant genomic landscape of the region, generating 833 639 to 3 259 361 contigs across eight metagenomes. GC content varied between 40.5% and 46.1%, with the highest values in 2018 on the W° side (Table S5). The majority of reads was from bacteria (92.3 ± 2.9%), followed by eukaryotes (5.9 ± 0.6%), while archaea and viruses were < 1% (0.85 ± 0.09% and 0.83 ± 0.04%, respectively). The eukaryote count included multicellular eukaryotes likely from free DNA in the water column (Fig. S2A, Table S6). After removing these, the primary eukaryotes were Chlorophyta (from 13.73% to 26.59%), with *Micromonas* sp. representing the dominant genus, followed by fungal Ascomycota and heterokont Perkinsozoa with a dominance of Sordariomycetes and *Perkinsus* spp., respectively.

Bacterial communities were similar between sites, dominated by Alpha- and Gammaproteobacteria, and Bacteroidetes (Fig. S2B, Table S6). Within Alphaproteobacteria, Pelagibacterales (*Candidatus* Pelagibacter ubique, SAR11) and Rhodobacterales (*Octadecabacter arcticus*) were predominant. Gammaproteobacteria were mainly represented by *Paraglaciecola psychrophile* (Alteromonadaceae), unclassified Spongiibacteraceae (Cellvibrionales) and Oceanospirillales (*Reinekea forsetii*). Bacteriodetes featured *Polaribacter* and *Formosa* as the dominant genera.

The archaeal community was mostly Euryarchaeota, with unclassified Euryarchaeota and Natrialbales (mostly *Natronolimnobius*) prevailing, alongside *Nitrosopumilus* sp. (Thaumarchaeota; Fig. S3A, Table S6). The viral communities were similar in 2018 on both sides, but distinct in 2017 (Fig. S3B, Table S6), predominantly consisting of unclassified and uncultured viruses, followed by known viruses, with a prevalence of *Cyprinivirus*, *Prasinovirus* and *Prymnesiovirus* (Phycodnaviridae).

### Metabolic potentials of the Arctic microbiome

**Figure 3:**
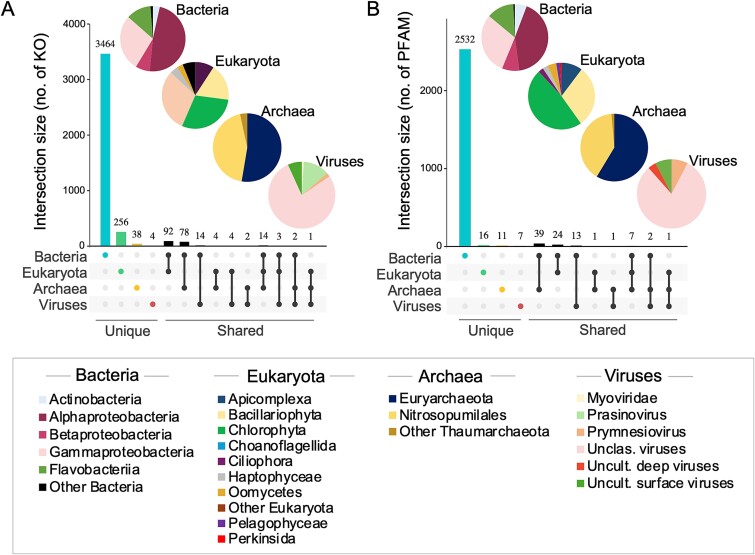
**Unique and shared KO and PFAM among the entire microbial community**. UpsetR shows the total number of different (**A**) KOs and (**B**) PFAM identified in each lineage, with unique and shared genes between the four lineages among all assembled metagenomes. Pie plots show communities composition of each lineage where KOs or PFAM were identified. A corresponding table for each KO and PFAM present in each lineage is in Table S7.

The metabolic potential of microbial communities was evaluated from assembled metagenomes, revealing 3979 distinct functional genes from KEGG Orthology (KOs; Fig. 3A, Table S7). Of these, 3464 KOs (~92%) were unique to bacteria, while eukaryotes accounted for 6.4%, archaea 3.4% and viruses 0.75%. Among bacteria, Alphaproteobacteria were the most prevalent (48%), followed by the Gammaproteobacteria (28%). Eukaryotic KOs were primarily from Choanoflagellida (30%) and Chlorophyta (29%), linked to the model reference species *Monosiga brevicollis* and *Micromonas pusilla*, respectively. Most archaeal sequences were unclassified Euryarchaeota (53%), and Nitrosopumilales (44%), closely matching *Candidatus* Nitrosopumilus sediminis. Among viruses, 78% of KOs were from unclassified viruses.

**Figure 4:**
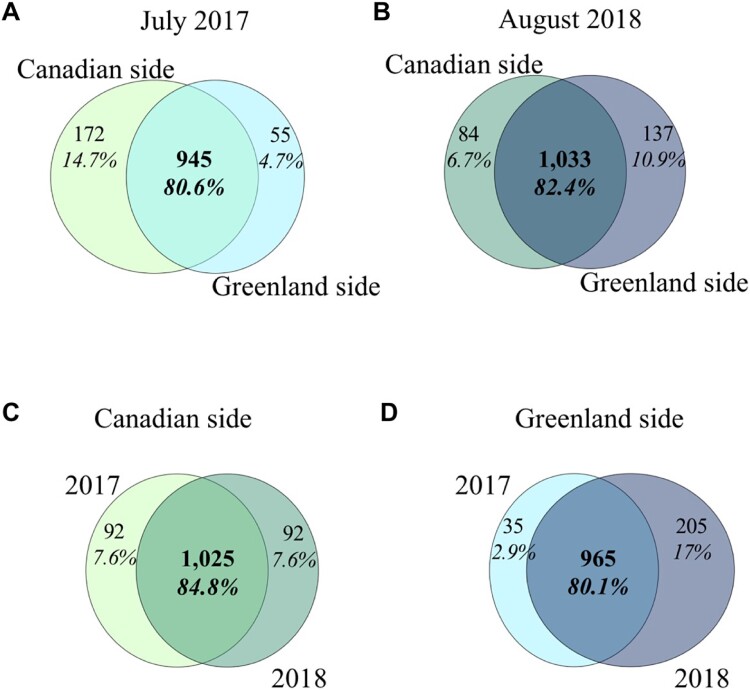
**Venn diagram of unique and shared COGs between all samples and all microbial communities.** The number of unique and shared COGs among assembled metagenomes are indicated. Comparison of number of COGs between sides of northern Baffin Bay only (**A**) in 2017 and (**B**) in 2018; and between years only (**C**) on Canadian side and (**D**) on Greenland side. A corresponding table for COG present in each lineage is in Table S7.

Additionally, 2532 distinct protein family domains (PFAM) were identified in Bacteria (Fig. 3B, Table S7), with Alpha- and Gammaproteobacteria being the most common, especially from *Candidatus* Pelagibacter and unclassified Porticoccaceae. In the protists community, PFAMs were mainly from Chlorophyta, particularly *Micromonas* sp. (Mamiellophyceae), while diatom (Bacillariophyta) PFAMs were primarily associated with *Thalassiosira* sp. (Mediophyceae). In archaeal and viral communities, most PFAMs were from Euryarchaeota and unclassified viruses, respectively. A total of 1291 different proteins were identified in the Cluster of Orthologous Groups of proteins (COGs), with just over 80% shared between sides and years (Fig. 4, Table S7).

### Metabolic pathways in protist communities

**Figure 5:**
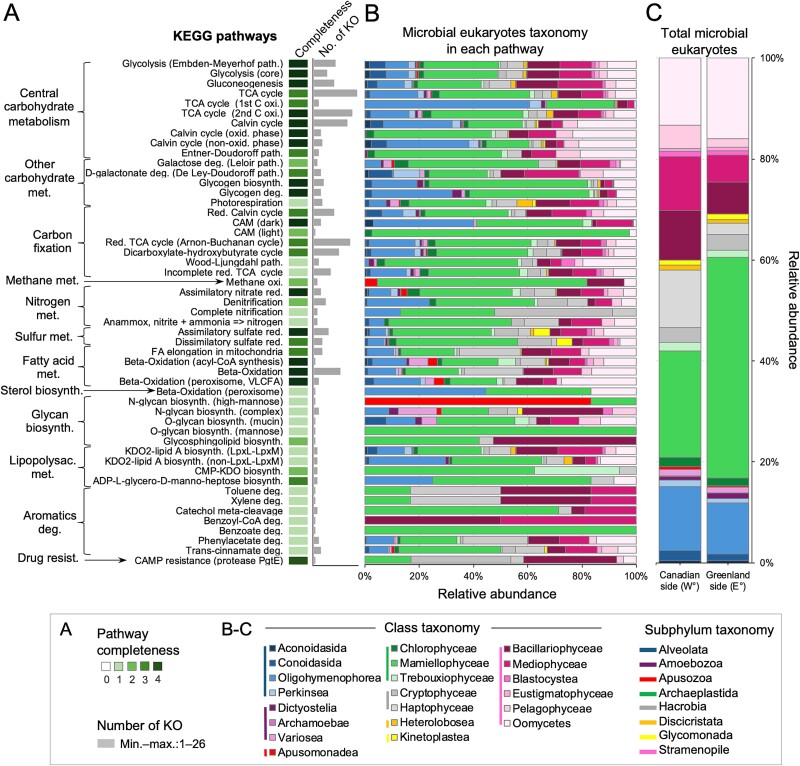
**Completeness of metabolic pathways among all metagenomes showing marine microbial eukaryotic by taxonomic affiliation.** (**A**) KEGG pathways, pathway completeness is indicated by intensity of the color: Absence of the pathway – 0; less than half complete – 1; half complete – 2; more than half complete – 3; and complete pathway – 4. The compilation of completeness of pathways and assigned taxonomy is in Table S8. (**B**) Proportions of the class of the retrieved genes in the pathways. (**C**) Comparison of the proportions of subphyla found on the two sides, from metagenomes.

KO numbers corresponding to microbial eukaryotic taxonomy as used in the KEGG database were retrieved (Fig. 5) to specifically investigate potential for the microbial eukaryotic community. Chlorophyta and Bacillariophyta (diatoms), were the most prevalent taxa within the community, and had the greatest diversity of KO genes within each KEGG pathway, except for two pathways. These exceptions were: (i) "complete nitrification" (M00804; Table S8), where no genes in the pathway were found in diatoms; and (ii) "Benzoyl-CoA degradation" (M00541) with no KO found in the Chlorophyta. Several KEGG pathways were found to contain all their constituent KOs, indicating complete metabolic pathways within the community. These pathways included those involved in central carbohydrate metabolism, carbon fixation, nitrogen, and sulfur metabolisms, including the “assimilatory nitrate reduction” (M00531) and the “assimilatory sulfate reduction” (M00176), as well as fatty acid metabolism. Protists classified within Mamiellophyceae, Bacillariophyceae, Mediophyceae, and Oomycetes, possessed KOs involved in the degradation of aromatic compounds.

### Metabolic prediction in bacterial and eukaryotic MAGs

**Figure 6:**
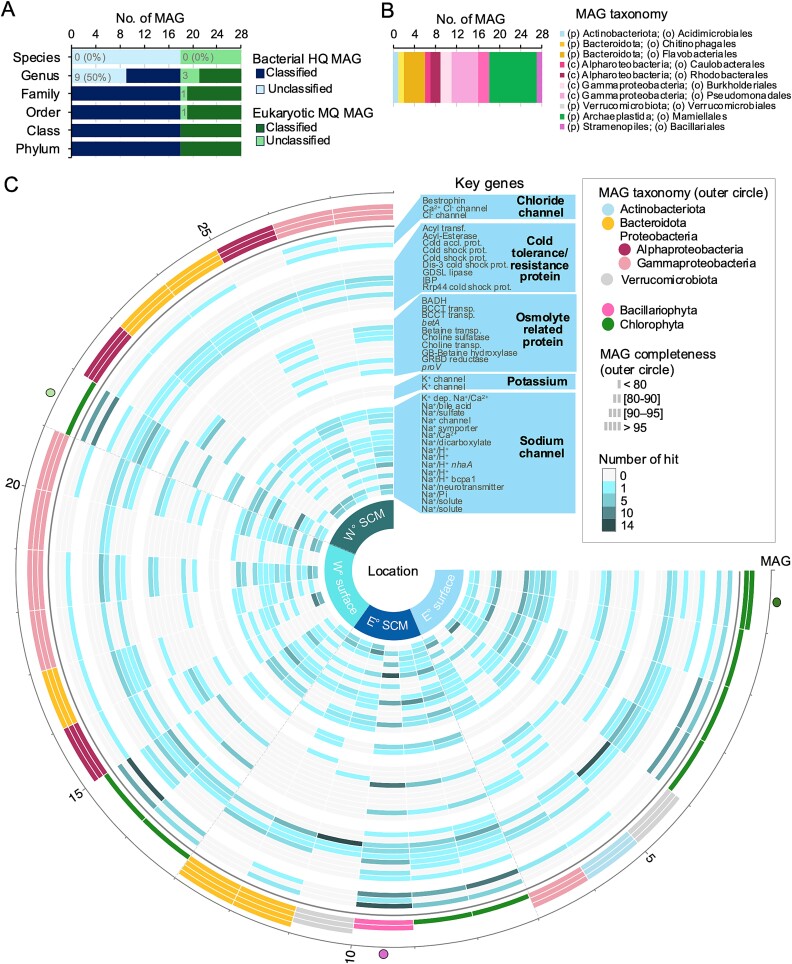
**High-quality bacterial and medium-quality eukaryotic MAGs and their selected functional annotations.** (**A**) Bar plot represents the total number of MAGs and the number having known and unknown classifications. (**B**) Bar plot showing the taxonomy at the lowest level for each MAG. (**C**) Circular heatmap of selected key genes present in each MAG. Colors in the outer circle represent the taxonomic affiliations of each MAG (phylum and class levels). Colors in the inner circle represent the sampling site: Either on the Canadian side of the North Water (W°) or on the Greenland side (E°). Depth of sampling is referred as surface and subsurface chlorophyl maximum (SCM). The three selected MAGs (see Fig. 7) are indicated by a colored circle outside the outer circle of the circular heatmap. A corresponding table with detailed information about each MAG is in Table S9 and a table for each gene present in each MAG is in Table S10.

The MAG assemblies yielded 18 high-quality (contamination: <15% and completeness: >90%) bacterial bins and 10 medium-quality (contamination: <15% and completeness: >50%) eukaryotic bins, which were from six different phyla (Fig. 6A-B). Nine bacterial MAGs and two chlorophyte MAGs remained unclassified at the genus level (Fig. S4, Table S9). Key metabolic genes related to freezing resistance and cold acclimatization were identified in the MAGs (Figs. 6C, Table S10). Ice-binding proteins (IBP, PF11999), were present in seven MAGs, including three eukaryotic (MAG-4, −8, and −22) from Chlorophyta and four bacterial (MAG-6, −17, −19, and −21) from Actinobacteriota, Bacteriodota, and Proteobacteria. The acyl-esterase protein (PF13839), linked to antifreeze functions, was only found in eukaryotic MAGs (MAG-10 and -15), while the GDSL-like lipase/acylhydrolase (PF13472) was present in almost all MAGs, except for MAG-20, a *Porticoccus* sp., which was 91.83% complete (Table S9). Regarding the analogous acyl-esterase, an acyl transferase (PF07779) was identified in eukaryotic MAGs affiliated with Chlorophyta and Bacillariophyta (MAG-1, −2, −8, −9, −15, and −22).

Given the fluctuating salinity conditions of Arctic surface waters, we examined inorganic ion transport and osmolyte-related proteins (Fig. 6C). Voltage-gated chloride channels (PF00654) and bestrophin (PF01062) were the most retrieved chloride transport PFAMS. Sodium (Na^+^) and hydrogen (H^+^) exchangers were frequently found, while potassium (K^+^) channels (PF01007 and PF17655) were retrieved in nine MAGs, predominantly eukaryotes MAG-3, −4, −8, −9, −10, −14, −15 and a single bacterial MAG (MAG-12). The betaine/carnitine/choline transporter (PF02028) was the most retrieved osmolyte-related protein, with Chlorophyta MAGs showing more osmolyte production genes than the Bacillariophyta MAG-10 (Table S10).

### Central metabolic cycles and energy metabolism revealed in MAGs

Genes associated with photosynthetic processes, including photosystem I and II (PSI and PSII) reaction centers, pigments and chlorophyll biosynthesis were identified in all eukaryotic MAGs (Fig. S5). Among the six genes related to PSI and PSII, *psaI* (TIGR03052) was only detected in MAG-10 (Bacillariophyta). Most coding DNA sequences (CDS) of MAG-10 were classified based on the model diatom *Phaeodactylum tricornutum* genome (Table S11). The genes *psaO, psb28,* and *psbM* (TIGR03059, TIGR03047, and TIGR03038, respectively) were identified among all eukaryotic MAGs classified to Chlorophyta, within the genera *Micromonas* and *Bathycoccus*, both in the Mamiellophyceae. Additionally, the gene coding for a light-dependent protochlorophyllide reductase (LPOR, TIGR01289) was detected in seven of the eukaryotic MAGs (MAG-1, −2, −8, −10, −14, −15, and −22). We found genes involved in the central metabolism cycles of methane, nitrogen, and sulfur, several were in all MAGs, such as a coenzyme F420 hydrogenase (hdr, K03388), a nitrate/nitrite transporter (narK, K02575), a nitrite reductase (NADH) large subunit (nirB, K00362), a sulfite reductase beta subunit (dsrB, K11181) and a sulfate adenyltransferase (sat, K00958; Fig. S5, Table S10).

### Degradation capacity revealed in bacterial and eukaryotic MAGs

Genes involved in multiple degradation pathways were detected in the 28 MAGs (Fig. S6, Table S10), including pathways for chlorinated and aromatic compounds, and other organic molecules. The most prevalent genes for degrading chlorinated compounds were S-hydroxymethyl glutathione dehydrogenase (*adhC*, K00121) and alcohol dehydrogenase (*exaA*, K00114). For aromatic compound degradation, two acyl-CoA dehydrogenases (*acd*, K00249 and *dcaA*, K06446) were widespread across all MAGs. Organic molecule degradation genes were found in several eukaryotic MAGs, with an alpha-amylase (*amyA*, K01176) mostly prevalent in MAG-3, −8, −9 and − 22; and a sialidase-1 (*neu1*, K01186) in MAG-1 and -2.

**Figure 7:**
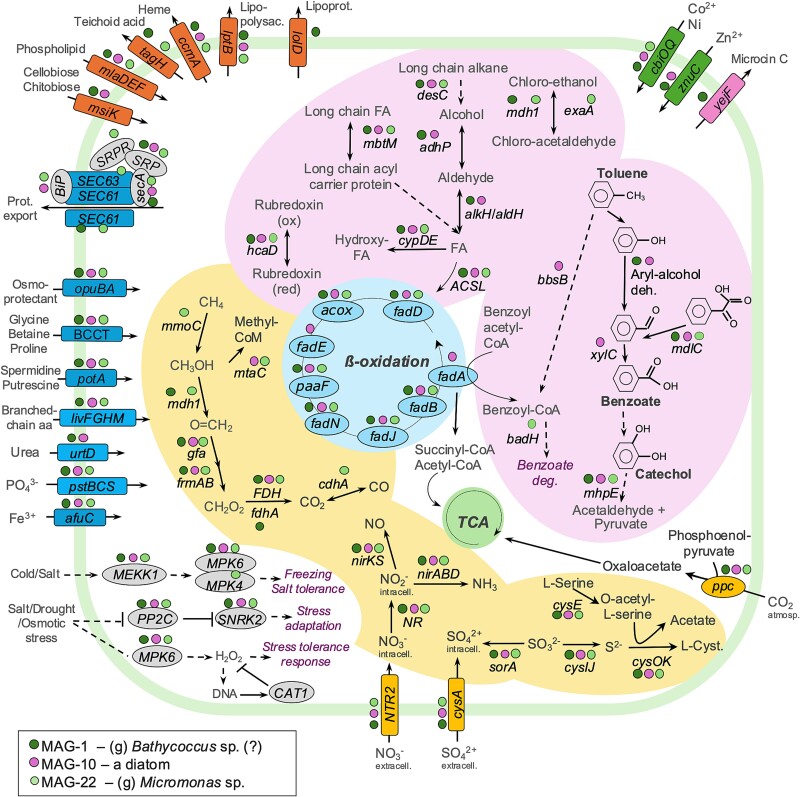
**Cell schematic diagram of key genes found in medium-quality eukaryotic MAGs.** Colored dots indicate predicted pathways and corresponding proteins in each eukaryotic genome to demonstrate the comparison between the three selected MAGs. The predictions are based on KEGG annotation. A corresponding table with detailed information about each MAG is in Table S9. For the corresponding table for proteins and pathways see Table S12. Acronyms: Tricarboxylic acid cycle – TCA. Diatom pathways were derived from the *Phaeodactylum* genome. see main text.

Three eukaryotic MAGs were selected for further analysis based on their completeness, low contamination, novelty, and diverse key genes related to salt and cold tolerance, osmoregulation, protein transport, nutrient uptake, methane oxidation, and potential hydrocarbon degradation (Fig. 7, Table S12). All three selected algal MAGs possessed the gene for phosphoenolpyruvate carboxylase (*ppc*, K01595), essential for oxygenic carbon fixation. The three algal MAGs also had genes for extracellular nitrate and sulfate uptake. Only MAG-22, whose annotation was based on *Micromonas commoda*, possessed genes for methane oxidation to methanol by methane monooxygenase *mmoC* (K16161), methanol to formaldehyde by methanol dehydrogenase (*mdh1*, K14028), and CO_2_ to CO by carbon -monoxide dehydrogenase *cdhA* (K00192). The MAP kinase signaling cascade pathway genes related to cold/salt tolerance and stress response were present in the three algal MAGs, including MEKK1, MPK4 and MPK6 (K04416, K20600 and K14512, respectively). Moreover, several genes encoding a complex of protein exporters were present in the three algal MAGs, whose annotations were based on CDS from *Bathycoccus prasinos*, *Phaeodactylum tricornutum*, and *M. commoda* genomes (Table S11). This complex of protein exporters includes the subunit of the protein SEC61, BiP, SRPR, SRP, and secA (K10956, K09490, K13431, K03106, and K03070; Fig. 7). The detailed analysis revealed genes for beta-oxidation of fatty acid and hydrocarbon degradation in all three algal MAGs. MAG-1 and -22 (Chlorophyta) possessed genes for chlorinated compound degradation (*mdh1* and *exaA*), while only MAG-10 (Bacillariophyta) possessed the benzoylsuccinyl-CoA thiolase (*bbsB*, K07550), and the benzaldehyde dehydrogenase (*xylC*, K00141) genes for toluene degradation.

## Discussion

### Environment selects microbial communities

Given the rapid changes in the Arctic, we assessed the genetic potential of microbial communities favored under specific open water conditions to infer ecosystem sustainability across the Arctic [38]. Interannual differences were consistent with seasonal effects, including light availability [5] and temperature (Fig. 2). Exacerbating differences between years may have been, the earlier collapse of the Nares Strait ice bridge in 2017 compared to 2018 [5, 39], which would have resulted in more Arctic pack ice flowing south [40]. This increased ice export to Baffin Bay would have generated colder, less saline waters on the Canadian side in 2017 (Table S1). The interactions between seasonal advance and ice bridge collapse on species composition warrants further study.

### Protist communities

Arctic restricted pico- and nanophytoplankton species are common in the Arctic [5, 7], suggesting a pan-Arctic species pool as a source for recruitment and success determined by local conditions. Arctic species and ecotypes of *Micromonas* (Chlorophyta), *Thalassiosira*, *Chaetoceros* (Mediophyceae), and *Strombidium* sp. (a ciliate; Figs. 2, 5, S2A, Table S4), are ubiquitous with pan Arctic distributions [7, 10, 34] making them key sentinels and targets for ongoing monitoring. Consistent with previous findings for mid-August [5], *Micromonas* spp. were prevalent on the E° side, while small diatoms, like *Chaetoceros* spp. prevailed on the W° side (Table S4).

The amplicon data retrieved greater microbial eukaryotic species diversity compared to metagenomic data partly due to a larger 18S rRNA database and limited sequencing depth in metagenomes, which primarily captures abundant organisms. For example, species-level identification from 18S rRNA indicated *Micromonas polaris* dominated the *Micromonas* OTUs (9279 reads; Table S4) and we also successfully assembled a *M. commoda* MAGs (Table S9). However, amplicon data is not quantitative due to primer bias affecting some stramenopiles and high gene copy numbers in alveolates, especially dinoflagellates and ciliates [41]. The source of nucleic acids can have an effect with 18S rRNA gene results influenced by the number of 18S rRNA gene copies in the genome of the cell, and rRNA results biased towards cells with more ribosomes.

Metagenomic approaches avoid primer biases but face challenges in quantifying microbial eukaryotes due to genome size differences and limited reference genomes. Identifying uncultivated microbial eukaryotes in metagenomes remains difficult. A recent massive sequencing effort with 280 billion reads from Tara Oceans recovered 265 photic zone MAGs, but only 3% could be assigned to the genus level, the most common genera were *Micromonas*, *Bathycoccus*, other chlorophytes, a diatom and haptophyte [42]. In comparison, nine of our 10 MAGs were *Micromonas* or *Bathycoccus*, along with a single diatom MAG. Overall, over 20% of the genes in our MAGs could not be classified taxonomically at any level (Fig. 6A). Metagenomic data was somewhat biased toward larger metazoans, as seen in the high occurrence of genes from Annelida, Arthropoda, Porifera (Fig. S2A), in keeping with free DNA persisting in marine waters, but species identification of these requires specific primers for any given group [43]. In addition, for *in situ* transcriptional and pathway confirmation, metagenomics is limited, and multi-omics approaches are needed.

### Bacterial, archaeal, and viral communities

Marine bacteria remain largely uncultured, though common groups can be classified using 16S rRNA [44], precise functional genomic information for most marine bacteria is still limited [45], despite the efforts to increase available genomes and transcriptomes [46, 47]. The assembled metagenomes revealed that Proteobacteria, followed by Bacteroidetes, dominated bacterial communities on both sides (Fig. S2B). The most abundant bacterial lineage in our study was *Candidatus* Pelagibacter ubique (SAR11, Alphaproteobacteria) cluster, which is ubiquitous [48], yet diverse, with potential unique clades in the Beaufort Sea [12]. Further analysis is required to determine if there are ecotypes specific to this *Pikialasorsuaq* region. The psychrophilic bacterium *Octadecabacter arcticus* (Alphaproteobacteria) found in *Pikialasorsuaq* is restricted to Arctic waters, while its sister species *O. antarcticus* occurs in Antarctica and Southern Ocean pack ice [49, 50] and was not found. Another species in the dataset, *Reinekea forsetii*, is found attached to algae, thriving under low phosphate conditions and may be competitive during spring phytoplankton blooms [51]. Actinobacteria, Firmicutes, Planctomycetes and Verrucomicrobiota were less abundant in our dataset in keeping with reports that they are rare taxa in polar environments [52].

The metagenome archaeal and viral reads represented <1% of the total microbiome, reflecting the difficulty in assigning reads to groups with few representatives in databases and potential loss of viral particles during successive size filtration with a minimum pore size of 0.2 *μ*m. Thaumarcheaota (*Nitrosopumilus* sp.) were previously reported in *Pikialasorsuaq* [14] and other Euryarchaeota were found in specific water masses [13]. Depending on season and location, archaea are reported to make up 1%–17% of the picoplankton in surface waters around Antarctica [53] and 1%–25% in the Arctic from samples collected in Amundsen Gulf and the Beaufort Shelf (Eastern Canadian Arctic) [54, 55]. In the present study, unclassified Euryarchaeota dominated the archaeal community (Fig. S3A), consistent with this group being dominant archaea in the other areas of the Arctic [56], where they could potentially be involved in nitrogen cycling [57]. Additionally, most viruses in the metagenomes were unclassified, except for Phycodnaviridae, with *Prasinovirus* the most abundant, followed by *Prymnesiovirus* (Fig. S3B). Phycodnaviridae use algae as hosts from both fresh and marine waters [58], and potential hosts *Micromonas* and *Phaeocystis* are both common in the Arctic and *Pikialasorsuaq* in particular [15].

### Protists capable of cold/salt resistance/tolerance

The 10 microbial eukaryote MAGs carried genes involved in inorganic ion transport, osmolytes, photosystems and key antifreeze proteins, such as IBP, acyl-esterase and cold-shock domain (CSD), similar to those reported in an ice-associated Pelagophyte [27, 28, 59]. These proteins were found in both Chlorophyta (MAG-1 to −4, −8, −9, −14, −15 and − 22) and the Bacillariophyta (MAG-10) MAGs (Fig. 6C). The nine Chlorophyta MAGs belonged to the Mamiellophyceae, within the genera *Micromonas* or *Bathycoccus*, which are the dominant picophytoplankton species in the Arctic Ocean [15]. The diatom MAG-10, with only 85.47% completeness, lacked 18S rRNA genes, but was likely one of the Arctic *Chaetoceros* spp. detected in the amplicon survey (Table S4). Commonly seen in microscopy studies, *Chaetoceros gelidus* in the *C. socialis* complex [60, 61] and *Chaetoceros neogracilis* are effectively indistinguishable at the 18S rRNA gene level [62]. Both species are reported as dominant summer species in the Arctic, including in the *Pikialasorsuaq* region [30, 63]. Unfortunately, no genomes of these species have been published to date and a related species *Chaetoceros tenuissimus* remains in a draft state [64] and is not in the KEGG or KOG databases. For these reasons, we based the annotation of MAG-10 on the model diatom *Phaeodactylum* with 64% of the CDS and *Thalassiosira* with 21% of CDS (Table S11). The higher proportion of *Phaeodactylum* coincides with its current position as the most established model diatom used in molecular research [65].

IBP were initially identified in the ice-associated diatom *Fragilariopsis cylindrus* [66, 67], and subsequently found in ice-algae, including diatoms, prymnesiophytes, prasinophytes, chlorophytes [68], and pelagophytes [27, 28]. IBPs are also widespread in phytoplankton that live in ice-associated regions [69] and are absent in temperate marine species, suggesting their necessity for survival in ice-influenced waters and sea-ice. The CSD family was widespread and particularly common in the metagenomes. CSD proteins bind to DNA and function as a regulator of various cellular functions, such as transcription, translation, and protein folding. It is involved in many biological processes, including low-temperature adaptation, cellular growth, gene regulation, and stress response [70].

We identified genes involved in inorganic ion transport (Fig. 6C), essential for regulating osmolarity in the fluctuating salinity of Arctic surface waters, associated with ice melt and freeze-up. Polar algae must be adapted to conditions near the freezing point of seawater, employing metabolic strategies such as cold-shock and antifreeze proteins and modifications to the photosynthetic electron transport chain to function at low temperatures [71]. In plants and animals, calcium ions act as the primary regulator of the initial responses to osmotic pressure [72, 73], and potassium and calcium channels, are thermosensitive and are thought to detect osmotic shock [74]. Numerous genes involved in light capture (e.g. *psa* and *psb*), were retrieved among eukaryotic MAGs (Fig. S5). The PSI and PSII reaction centers are ancient, dating to the origin of photosynthesis, and found in present day chloroplasts [75]. Light availability is highly variable in the polar environment, and fluctuations in the underwater light climate strongly influence photosynthetic productivity [76, 77]. Even under the unique light conditions of Arctic summers, with 24-hour daylight and low solar angles, photosynthetic microorganisms still need and employ photoprotective mechanisms, and diatoms are particularly well-adapted to varying light conditions [78]. In addition to light regime induced photoacclimation, temperature and salinity shifts can induce this mechanism in both chlorophytes and diatoms by increasing PSII proteins and photoprotective pigments [79, 80].

### Potential hydrocarbon degradation by algae

Metagenomes and MAGs revealed hydrocarbon degradation genes in the protist community (Figs. 7, S6), indicating potential for polar marine phytoplankton to biodegrade hydrocarbons. Although not previously reported in polar microalgae, freshwater green algae (*Selenastrum*, *Scenedemus*, and *Chlorella*) and marine algae (*Chlamydomonas*, *Dunaliella*, and *Nitzschi*a) can degrade polycyclic aromatic hydrocarbons (PAHs) [81, 82]. More recently, the marine chlorophyte *Ostreococcus tauri* has been shown to have the same capacity [83]. These findings suggest that in addition to the their pivotal role as primary producers, microalgae likely contribute to PAH degradation in both marine and freshwaters [81]. Our findings suggest that *Pikialasorsuaq* phytoplankton may also be involved in hydrocarbon cycling by reducing PAH bioavailability though degradative pathways and by reducing toxicity through the production of exopolysaccharides [84, 85]. These polymers mediate the uptake of contaminants on the cell surface and/or their complexation into less bioavailable forms [85].

Marine cyanobacteria synthesize hydrocarbons [86] and their alkanes/alkenes are believed to play a prominent role in the marine alkane cycle of the upper ocean [87, 88]. Obligate hydrocarbon-degrading bacteria are found in waters without significant levels of petroleum pollution [87] and in the absence of fossil hydrocarbons [89] indicating that these organisms would use an alternate hydrocarbon source. Cyanobacteria, which are rare to absent in polar waters [90] would not be a source of hydrocarbons, however, several eukaryotic phytoplankton including *Chaetoceros* sp., *Thalassiosira* sp. [91], and dinoflagellates, such as *Amphidinium* sp. [92] also synthesize hydrocarbons. These phytoplankton lineages were the most prevalent in our amplicon and metagenomics data (Figs. 2, S1, Table S6). Marine algae can also produce other hydrocarbons including isoprene [93, 94], which may sustain hydrocarbon-degrading bacterial populations in fossil-oil free environments.

### Potential methane aerobic utilization by the pelagic community

Metagenomes and MAGs revealed methane oxidation pathways in Arctic Apusomonadea, Mamiellophyceae, Bacillariophyceae and Oomycetes (Figs. 5, S5). Further analysis of the three selected eukaryotic MAGs revealed that one of them (MAG-22, *Micromonas*) possessed *mmoC* (Fig. 7, Table S12), a component of methane monooxygenase. This indicates the potential for aerobic methane utilization in the *Pikialasorsuaq* pelagic zone, a phenomenon previously observed in specific areas where cold methane-rich fluids leaked from subsurface reservoirs, which reached the seafloor and water column in marine environments [95, 96]. Sea-ice freezing and melting has been identified as a source of methane in the Arctic Ocean [97], where methane drawn from the atmosphere becomes trapped under ice in winter [98] suggesting that methane is a potential energy source [99] for both bacteria and algae in the oligotrophic surface mixed-layer during the dark winters. While aerobic methane oxidation by bacteria is well-documented, less is known about marine protists' role in methane oxidation, offering a possible explanation for the 'methane paradox,' where bacterial numbers may be insufficient to account for methane oxidation [100].

## Conclusion

Our shotgun metagenomic analysis of microbial communities from the two sides of *Pikialasorsuaq* revealed previously undocumented aspects of metabolic adaptation in polar environments. The community biosynthetic pathways reflected local adaptations to Arctic conditions that include low temperatures, freshening salinities, low nutrient concentrations and variable light levels within the upper mixed-layer. More surprising, was that both eukaryotes and bacteria in the microbial community have the genetic capacity for potential bio-attenuation of methane, as well as metabolising hydrocarbon compounds. In addition, we identified and addressed several gaps in marine microbial eukaryote reference data, providing direction future genomic and transcriptomic analyses of isolates from polar regions. The functional adaptations we uncovered contribute to an emerging understanding of how extremophiles may respond to environmental change, with implications for predicting ecosystem responses to climate change. These findings not only expand our knowledge of polar microbial ecology but also provide essential baseline data for future studies of marine ecosystem resilience in rapidly changing environments.

## Supplementary Material

ISME_Comm_suppl_materials_20250112_ycaf007

Supplementary_tables_metagenomes_NOW_20250106_ycaf007

## Data Availability

All amplicon results are in the NCBI GenBank Sequence Read Archive (SRA) under BioProject PRJNA662595 (GenBank: SRS7367825 to SRS7367873). Oceanographic data is available from Amundsen Science: Amundsen Science Data Collection. [2017–2018]. Processed data. Version 1. Archived at www.polardata.ca. Annotated assembled metagenomes data are available in IMG/M under the following accession numbers: 3300035184, 3 300 035 487, 3 300 037 191, 3 300 038 734, 3 300 038 375, 3 300 038 650, 3 300 039 223 and 3 300 039 232. Additonal details of all methods are provided in the Supplementary Materials Table S13.

## Code availability

This paper does not report original code. All code was adapted from the user manual of the software packages.
