# Supplementary material for: Shotgun metagenomics reveals the flexibility and diversity of Arctic marine microbiomes
Source: ISME Commun. 2025 Jan 21;5(1):ycaf007. doi: 10.1093/ismeco/ycaf007 (PMC11847657; doi:10.1093/ismeco/ycaf007)
Supplement: ISME_Comm_suppl_materials_20250112_ycaf007 [file isme_comm_suppl_materials_20250112_ycaf007.pdf]

**Shotgun metagenomics reveals the flexibility and diversity of Arctic marine microbiomes.**

Nastasia J. Freyria[1,2,3], Thais C. de Oliveira[3,4], Arnaud Meng[5,6], Eric Pelletier[6,7] and Connie Lovejoy[2,3]

[1]Department of Natural Resource Sciences, McGill University, Ste. Anne-de-Bellevue, Québec, QC, H9X 3V9, Canada

[2]Département de biologie, Québec Océan, Université Laval, Québec, QC, G1V 0A6, Canada

[3]Institut de Biologie Intégrative et des Systèmes Université Laval, Québec, QC, G1V 0A6, Canada.

[4]Centre d'Étude de la Forêt, Faculté de Foresterie, de Géographie et de Génomique, Université Laval, Québec, QC, G1V 0A6, Canada

[5]Institut Pasteur, Université Paris Cité, Metabolomic Core Facility, Paris, France

[6]Génomique Métabolique, Genoscope, Institut de Biologie François Jacob, CEA, CNRS, Université Paris-Saclay, Evry, France

[7]Research Federation for the study of Global Ocean Systems Ecology and Evolution, Paris, France

## Supplementary Method:

### Field sampling

Sampling was carried out during ArcticNet missions, onboard the research icebreaker *CCGS Amundsen* in late July 2017 and late August 2018 (**Table S1**). Samples were collected between Ellesmere Island, Canada, and the west coast of Greenland (**Fig. 1A**). To compare the two-opposite sides of *Pikialasorsuaq*, we selected two stations (Stn 101 and Stn 115), as part of a west to east transect along a line from -71.18 °W to -77.40 °W Longitude, and from 76.33 °N to 76.38 °N Latitude (**Table S1**). Surface (0-5 m) and subsurface of chlorophyll maximum (SCM, 25-37 m) depths were targeted (**Table S2**).

Seawater samples for nutrients, flow cytometry (Seapoint Sensors Inc.), and nucleic acids were collected using a Rosette system equipped with 12–L Niskin-type bottles and sensors as previously described (1). Water samples for nitrate ($NO_3^{2-}$), phosphate ($PO_4^{3-}$) and silicate ($SiO_4$) measurements were filtered through a 0.2 $\mu$m polycarbonate (PC) filters (AMD manufacturing) into acid-rinsed 15 mL Falcon tubes (Corning Inc.) and analyzed on board the ship using a Bran-Luebbe 3 auto-analyzer (2). The samples for nucleic acids were collected as in Freyria *et al.,* (3). Briefly within an hour of collection, ca. 6-8 L of seawater was filtered through 3 $\mu$m pore size 47 mm diameter PC filters, particulate material was collected into 0.3 $\mu$m Sterivex filters, preserved in RNAlater, and subsequently stored at -80 °C until analysis.

### Flow cytometry procedures and analyses

Flow cytometry samples were preserved with 90 $\mu$L of 25% glutaraldehyde added to 1.8 mL of seawater (final concentration of 1% v/v). The fixed samples were left for 30 min at 4 °C in the dark, then flash frozen in liquid nitrogen and stored at -80 °C until use. Bacterial and archaeal

cells, which are not distinguished with this method and are referred to total bacteria, were counted based on protocols in Marie *et al.,* (4) using a Accuri C6 flow cytometer (BD Biosciences) equipped with the Csampler and 14.7 mW 640 nm Diode Red Laser and 20 mW 488 nm Solid State Blue Lasers. Briefly, 200 $\mu$L of each sample were stained with 0.5 $\mu$L of Sybr Green I (Invitrogen) from a 1000X stock solution. Data acquisition was performed at a slow flow rate (14 $\mu L.min^{-1}$) for 5 min with 3 wash and 3 agitation cycles between each sample. To standardize counts and correct for salinity-related flow-speed variations, a known quantity of beads was added (BD Trucount Absolute Counting Tube, Fischer Scientific) to standards, and the cytometer was calibrated for each use. Eukaryotic pico- and nanophytoplankton were determined based on chlorophyll red fluorescence and side scattered light at 670 nm based on methods adapted (5, 6). Fluorescence and scatter properties of the unstained samples were measured using the BD Accuri, with data acquisition performed at fast flow rate (66 $\mu L.min^{-1}$) for 10 min with 3 wash and 3 agitation cycles between each sample. Data were processed with BD CSampler Software.

**Nucleic acid extraction and sample preparation**

DNA and RNA were extracted from the same filters using the All-Prep DNA/RNA Minikit (Qiagen) as described in Dasilva *et al.,* (7). Microbial eukaryotic community composition was determined by high-throughput 18S rRNA gene (sometimes referred to as rDNA) and 18S rRNA (rRNA) sequencing as in Freyria *et al.,* (3). Conversion of RNA to cDNA was carried out using the HighCapacity Reverse Transcription Kit (Applied Biosystems) following the manufacturer's suggestions. The V4 region of 18S rRNA in both DNA and cDNA samples was targeted as in Comeau *et al.,* (8) using eukaryotic specific primers (E572F/E1009R) coupled with MiSeq adaptors. All prepared samples were subjected to a nested PCR under conditions as in Comeau *et al.,*

(8). The PCR products were purified and quantified as in Freyria *et al.,* (3). The quality and the quantification of DNA content was verified using NanoDrop 8000 spectrophotometer (Thermo Fisher Scientific) as well as a Qubit fluorometer (Life Technologies) using the dsDNA BR Assay Kit (Invitrogen).

**Illumina MiSeq amplicon sequencing and analysis**

The amplicons were generated using the Illumina MiSeq at the plateforme d'analyses génomiques (IBIS, Université Laval, Québec, Canada). The resulting reads were processed as in Freyria *et al.,* (3). Briefly, sequence reads were quality checked, filtered and de-replicated using BBMerge v.37.36 (9). Chimeras were identified and removed using VSEARCH v.2.5 (10). Operational Taxonomy Unit (OTU) picking at >98% of similarity level was carried out using USEARCH v.10.0.240 (11) and MOTHUR v.1.41.1 (12). Taxonomic affiliation was determined using two curated 18S rRNA gene reference databases, Silva v.132 (13) and $PR^2$ v.4.1 (14).

**Illumina Solexa metagenomic sequencing and analyses**

For metagenomics sequencing, samples were extracted as above. Following quality checks, the metagenome samples were sent for library preparation and sequenced at Laboratoire de séquençage at Genoscope, CEA, Évry, France, using the Illumina Solexa Genetic analyzer as described previously (15). Metagenome sequence data were quality filtered using Trimmomatic v.0.36 (16), with default setting for paired-end Illumina data. Before assembling reads, paired-end reads were joined to make longer reads using the "*join_paired_ends.py*" script from QIIME package (17) with default setting. Megahit v.1.1 (18) was used to assemble paired-end joined and unjoined reads passing quality filtering. After assembly, all unassembled reads, which passed quality filtering,

were mapped back to assembled contigs to find unassembled reads using BBMap v.38.44 (9). Unmapped reads were concatenated with assembled contigs to generate fasta files for upload to IMG/M analysis pipeline (19). IMG/M pipeline was used for gene calling, functional annotation, and taxonomic affiliation of assembled contigs. Rarefaction and normalization of all metagenomes was done for comparing samples and to compare community composition in all samples. The gene count in each metagenome was normalized by hits per million genes based on the count of genes divided by the total number of reads multiplied by a million.

**Binning of metagenomes**

We performed metagenomic binning of each of the 8 metagenome assemblies individually to re-construct metagenome-assembled genomes (MAGs). To retrieve eukaryote bins we used EukRep v0.6.7 (20) to separate eukaryotic contigs from prokaryotic ones. Multiple binning tools, including MetBAT2 v2.15 (21), MaxBin2 v2.2.7 (22), and concoct v1.1.0 (23), were used for genome binning to increase the number of bins. Only contigs longer than 2000 bp were retained. The completeness and contamination level of all bacterial bins were assessed using CheckM v1.1.3 (24), and only bacterial bins with a contamination level below 15% and a completeness greater than 50% were kept. The quality of eukaryotic bins were verified with EukCC v2.1.0 (25). The replication of bacterial and eukaryotic bins was verified using dRep v3.2.2 (26). Taxonomic determination for the bacterial bins was done using the Genome Taxonomy Database Toolkit v2.1.0 (27). Functional annotation for all bins were done using MetaErg (28). The analyses were conducted using Compute Canada facilities and in-house computers.

**Statistics analyses**

All statistical analyses were carried out in R Studio v.1.4.1106. Spearman's rank-order correlation was performed and visualized using the *cor(), rcorr()* and *corplot()* functions from "Hmisc" and "corrgram" packages (29, 30). Constrained Correspondence Analysis (CCA) was computed using *cca()* function, to discriminate the different sampling stations according to the geographic location and environmental variables. The independent parameters that best explained variability in the CCA were selected using *ordiR2step()* and *envfit()* functions of the "vegan" package (31), by automatic forward selection, which selects variables to build optimal model with the highest adjusted coefficient determination. Unweighted Pair Group Method with Arithmetic Mean (UPGMA) was carried out on rarefied and normalized metagenome for each group of community (Bacteria, Eukaryota, Archaea and virus) based on the Bray-Curtis distance matrices using QIIME. Non-Parametric Multivariate Analysis of Variance (NPMANOVA) was used to test differences in composition between the two sides of *Pikialasorsuaq* and the two depths. NPMANOVA was performed by first calculating dissimilarity using *vegdist()* function from the "vegan" package.

**Table S1.** Station sampling date, location, and values for temperature, salinity and Chlorophyll *a* fluorescence (Fluo.) at each sampling depth: surface (Surf.), subsurface chlorophyll maximum (SCM) of 2017 and 2018. Bold values represent the lowest and highest values.

| **Date** | **Latitude (°N)** | **Longitude (°W)** | **Depth (m)** | | **Temperature (°C)** | | **Salinity** | | **Fluo. ($\mu$g Chl *a* $L^{-1}$)** | |
|---|---|---|---|---|---|---|---|---|---|---|
| | | | **Surf.** | **SCM** | **Surf.** | **SCM** | **Surf.** | **SCM** | **Surf.** | **SCM** |
| *Canadian side (W°- stn 101)* | | | | | | | | | | |
| 2017-07-24 | 76.38 | -77.40 | 2 | 37 | -0.04 | **-1.04** | **28.72** | 31.72 | **0.08** | 1.36 |
| 2018-08-26 | 76.38 | -77.40 | 5 | 35 | 2.21 | -0.62 | 31.92 | 32.61 | 0.29 | 0.61 |
| *Greenland side (E°- stn 115)* | | | | | | | | | | |
| 2017-07-27 | 76.33 | -71.20 | 2 | 25 | **4.45** | -0.89 | 31.63 | **33.57** | 0.23 | **2.11** |
| 2018-08-29 | 76.33 | -71.18 | 2 | 32 | 3.11 | 0.27 | 31.87 | 33.01 | 0.26 | 2.07 |

**Table S2.** Nutrient concentrations at each depth: surface (Surf.) and subsurface chlorophyll maximum (SCM) from both sides of the North Water in July 2017 and August 2018. Bold values represent the lowest and highest values.

| Side | Sampling date | Depth (m) | | Concentration in $\mu$M | | | | | |
|---|---|---|---|---|---|---|---|---|---|
| | | | | Nitrate | | Silicate | | Phosphate | |
| | | Surf. | SCM | Surf. | SCM | Surf. | SCM | Surf. | SCM |
| Canada | 2017-07-24 | 2 | 40 | n.d. | 1.02 | 2.88 | 5.48 | 0.53 | 0.67 |
| (West, W°) | 2018-08-26 | 5 | 40 | n.d. | 3.57 | 2.26 | **9.12** | 0.52 | **0.83** |
| Greenland | 2017-07-27 | 2 | 30 | n.d. | **5.32** | **1.28** | 5.16 | 0.44 | 0.71 |
| (East, E°) | 2018-08-29 | 2 | 30 | n.d. | **0.85** | 2.75 | 3.17 | 0.41 | **0.32** |

n.d. – not detected

**Table S3.** Flow cytometry cell enumeration: Eukaryotes in $10^4$ cells $mL^{-1}$); Bacteria in $10^6$ cells $mL^{-1}$. See **Fig. S1**.

| | Canadian side (°W) | | Greenland side (°E) | |
|---|---|---|---|---|
| | **2017** | **2018** | **2017** | **2018** |
| ***Picophytoplankton <3 µm*** | | | | |
| Surface | 0.32 | 3.28 | 14.7 | 5.99 |
| SCM | 2.83 | 6.09 | 33.3 | 8.51 |
| ***Nanophytoplankton >3 µm*** | | | | |
| Surface | 0.34 | 0.62 | 0.38 | 0.74 |
| SCM | 2.55 | 0.57 | 1.24 | 2.75 |
| ***Total Bacteria*** | | | | |
| Surface | 0.37 | 7.68 | 0.05 | 9.21 |
| SCM | 0.67 | 4.65 | 1.38 | 8.43 |

**Table S4 (xlsx, see separate compilation of excel sheets):** Table of OTUs in all samples and the corresponding taxonomy from **Fig. 2C**.

**Table S5.** Metagenomic sequencing and assembly of 8 metagenomes from both sides of Northern Baffin Bay of both years of sampling (July 2017 and August 2018).

| Year | Depth | Genome size (bp) | Nbr. Scaffold | Nbr. contig | Total length | Average bp | N50 bp | GC% | IMG Genome ID |
|---|---|---|---|---|---|---|---|---|---|
| *Canadian side (W°- stn 101)* | | | | | | | | | |
| 2017 | Surface | 119,088,561 | 7,409,167 | 1,660,259 | 1,078,011,823 | 649 | 706 | 41.7 | 3300037191 |
| | SCM | 102,876,015 | 5,526,179 | 1,177,993 | 749,328,237 | 636 | 687 | 42.4 | 3300038734 |
| 2018 | Surface | 354,650,250 | 17,400,537 | 3,558,066 | 2,159,684,930 | 607 | 622 | 45.3 | 3300039223 |
| | SCM | 413,330,403 | 13,236,522 | 2,587,230 | 1,556,885,729 | 602 | 608 | 46.1 | 3300039232 |
| *Greenland side (E°- stn 115)* | | | | | | | | | |
| 2017 | Surface | 80,248,839 | 3,467,985 | 833,639 | 534,344,561 | 641 | 694 | 40.5 | 3300035184 |
| | SCM | 176,373,857 | 5,362,633 | 1,364,202 | 849,539,283 | 623 | 649 | 41.0 | 3300035487 |
| 2018 | Surface | 405,058,950 | 10,312,359 | 2,234,058 | 1,439,932,202 | 645 | 679 | 40.9 | 3300038375 |
| | SCM | 530,394,972 | 13,626,032 | 3,259,361 | 1,995,169,613 | 612 | 619 | 43.3 | 3300038650 |

**Table S6 (xlsx):** List of taxonomy based on IMG/M and relative abundance, number of reads and hit per million gene in log scale for eukaryotes, bacteria, archaea and viruses community among the 8 metagenomes from **Figs. S2-S3.**

**Table S7 (xlsx):** See separate compilation of excel sheets. List of KEGG Orthology (KO), PFAM protein domain and Cluster of Orthologous Groups of proteins (COGs) found in assembled metagenomes within each lineage (archaea, bacteria, eukaryota and virus) from **Figs. 3 and 4**. KO, PFAM and COG were retrieved from the functional annotation performed by IMG/JGI.

**Table S8 (xlsx):** List of completeness pathways and assigned taxonomy present in all assembled metagenomes from **Fig. 5.** Pathway completeness is indicated by number: absence of the pathway – 0, less than half complete – 1, half complete – 2, more than half complete – 3, and complete pathway – 4. Number of counts and hit per million genes in log scale for each unique KEGG Orthology (KO)found within each taxonomy.

**Table S9 (xlsx):** See separate compilation of excel sheets. See separate compilation of excel sheets. Bacterial and eukaryotic metagenome-assembled genomes (MAGs) supplemental information.

**Table S10 (xlsx):** See separate compilation of excel sheets. List of bacterial and eukaryotic metagenome-assembled genomes (MAGs) and key genes from **Figs. 6, S5 and S6**. Each value represents the number of counts per gene in each MAG.

**Table S11 (xlsx):** See separate compilation of excel sheets. Number of coding sequences (CDS) of eukaryotic metagenome-assembled genomes (MAGs) and their proportion assigned to a taxonomy.

**Table S12 (xlsx):** See separate compilation of excel sheets. List of proteins and pathways present in the three selected eukaryotic metagenome-assembled genomes (MAGs) from **Fig. 7**.

**Table S13.** Gene calling details for the 8 assembled metagenomes. Number of KEGG Orthology (KO), PFAM protein domain and Cluster of Orthologous Groups of proteins (COGs) were after selection to 99% identification level

| Year | Depths | Gene count | RNA count | Genes with predicted function | KEGG count | KO count | PFAM count | COG count |
|---|---|---|---|---|---|---|---|---|
| *Canadian side (W°- stn 101)* | | | | | | | | |
| 2017 | Sur-face | 6,975,870 | 36,049 | 6,939,821 | 517,348 | 800,895 | 1,092,222 | 1,226,596 |
| | SCM | 5,210,954 | 25,556 | 5,185,398 | 426,238 | 659,978 | 866,244 | 999,121 |
| 2018 | Sur-face | 15,577,620 | 76,844 | 15,500,776 | 622,327 | 951,726 | 1,328,953 | 1,389,200 |
| | SCM | 11,608,907 | 50,671 | 11,558,236 | 666,928 | 1,002,709 | 1,377,829 | 1.480,751 |
| *Greenland side (E°- stn 115)* | | | | | | | | |
| 2017 | Sur-face | 3,314,874 | 16,550 | 3,298,324 | 329,729 | 500,792 | 638,508 | 737,640 |
| | SCM | 5,131,178 | 28,401 | 5,102,777 | 635,357 | 941,239 | 1,146,592 | 1,326,965 |
| 2018 | Sur-face | 9,541,441 | 62,874 | 9,478,567 | 764,743 | 1,132,061 | 1,455,463 | 1,635,765 |
| | SCM | 12,479,047 | 69,000 | 12,410,047 | 1,017,328 | 1,509,689 | 1,935,469 | 1,935,469 |

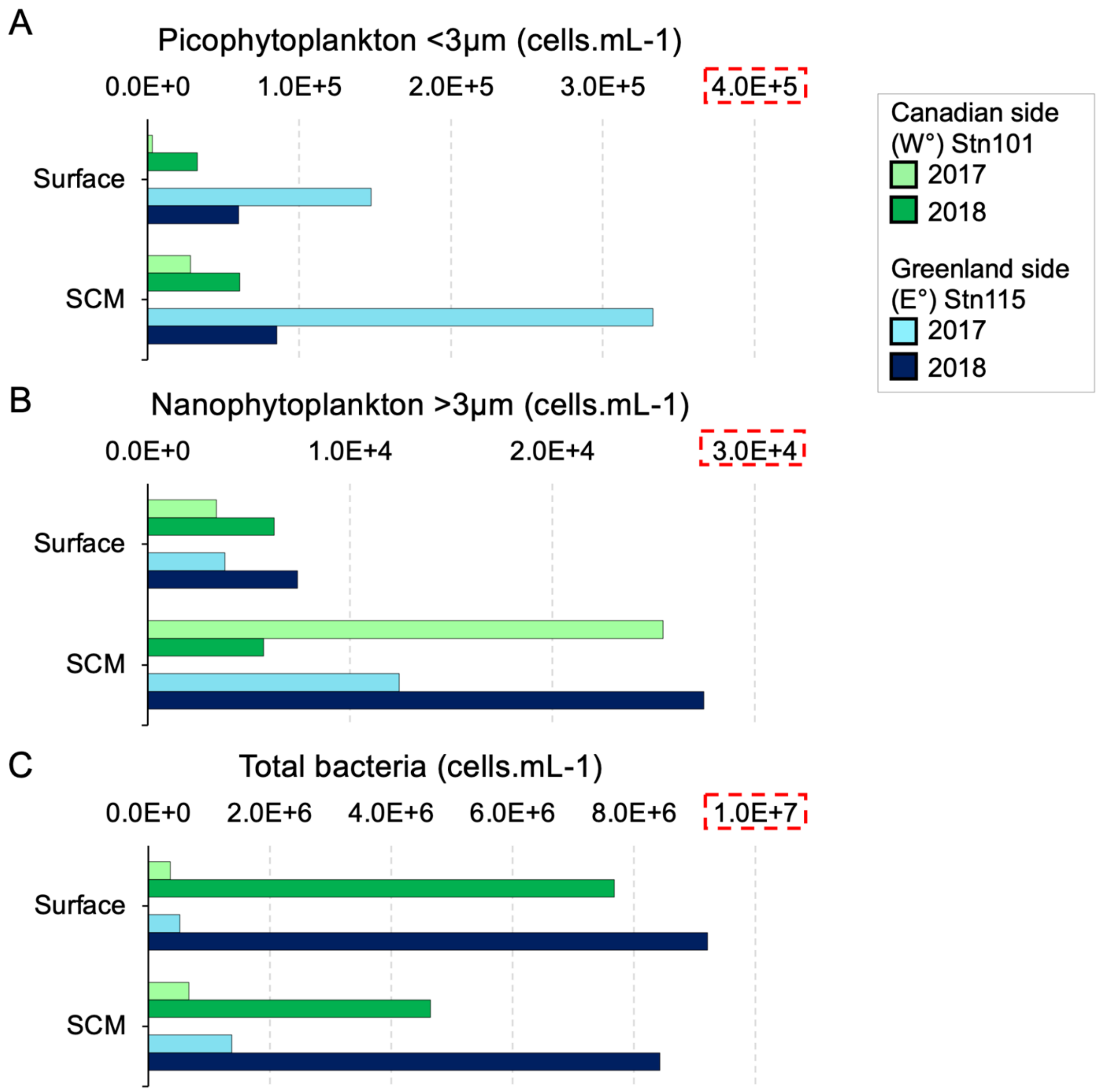


**Fig. S1. Flow cytometry cell concentrations.** Cell concentration (cells $mL^{-1}$) from the two years from surface and subsurface chlorophyll maximum (SCM) of **A)** picophytoplankton, **B)** nanophytoplankton and **C)** total Bacteria, including Bacteria and Archaea for each side.

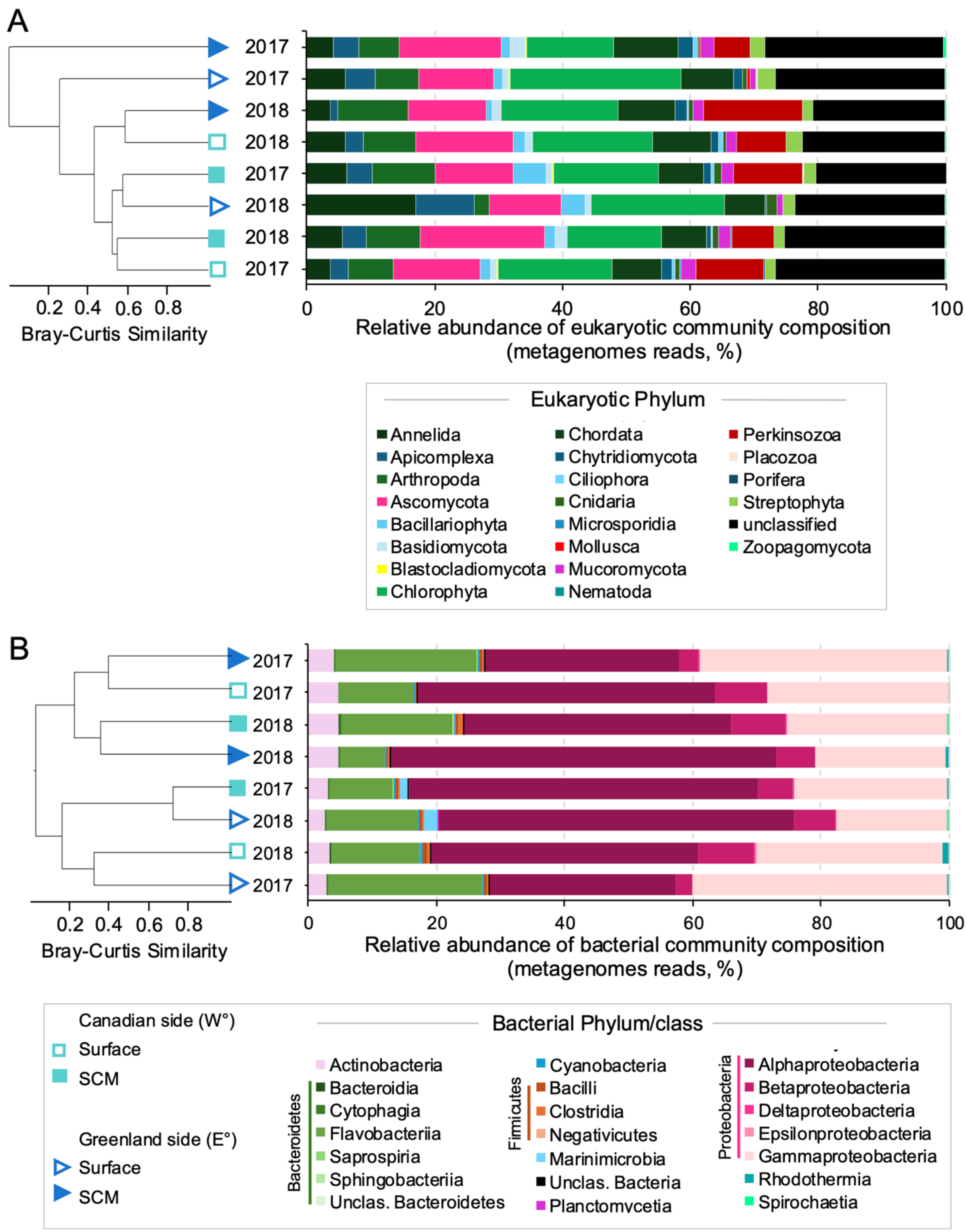


**Fig. S2. Relative abundance of Eukaryota and bacteria communities.** Community composition based on IMG/M taxonomic affiliation of metagenomic reads for **A)** total Eukaryota and **B)** bacterial communities. Unweighted Pair Group Method with Arithmetic Mean (UPGMA) was carried out based on the Bray Curtis distance matrices.

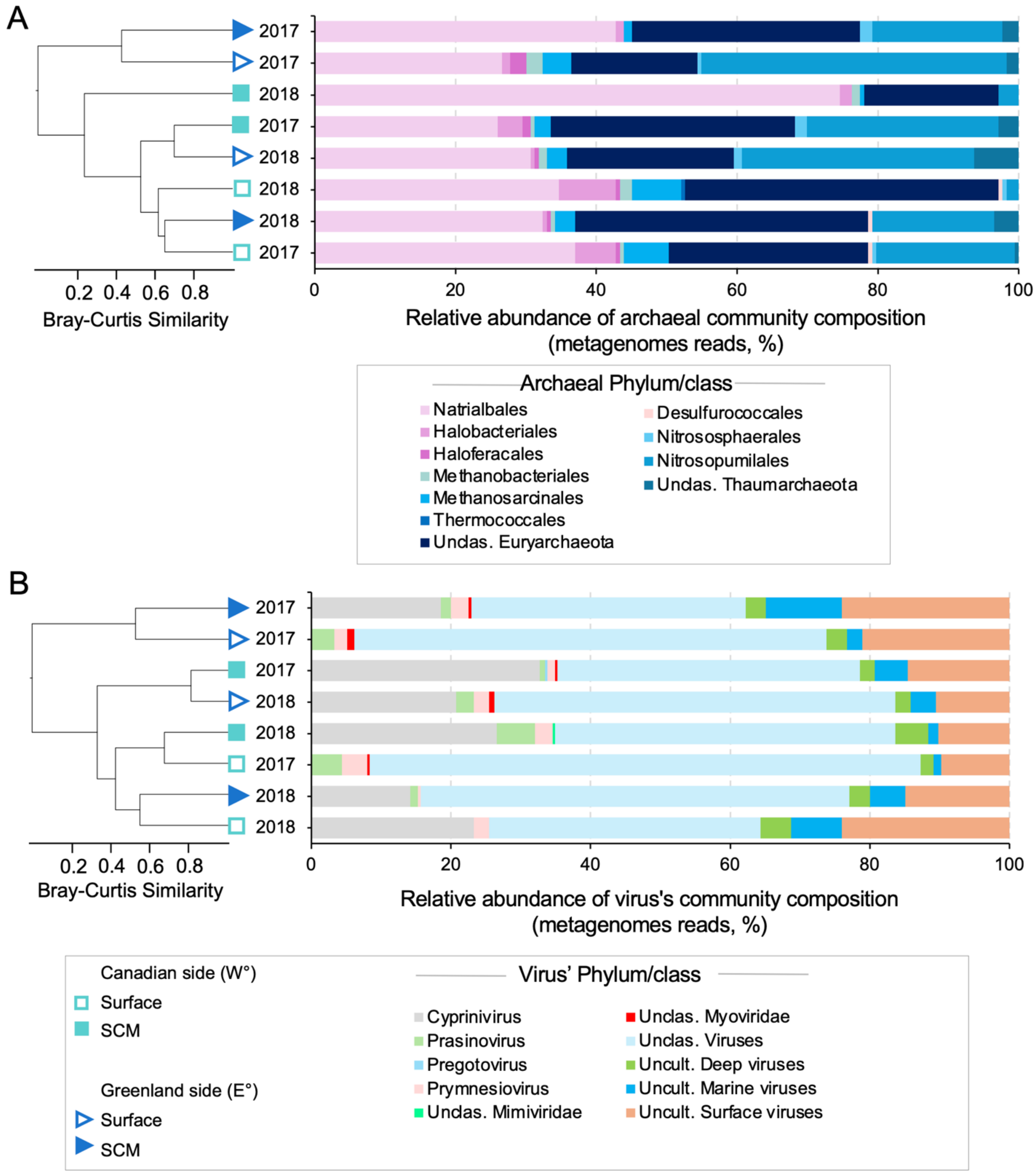


**Fig. S3. Relative abundance of Archaea and Viruses communities.** Community composition based on IMG/M taxonomic affiliation of metagenomic reads for **A)** Archaea and **B)** Viruses communities. Unweighted Pair Group Method with Arithmetic Mean (UPGMA) was carried out based on the Bray Curtis distance matrices.

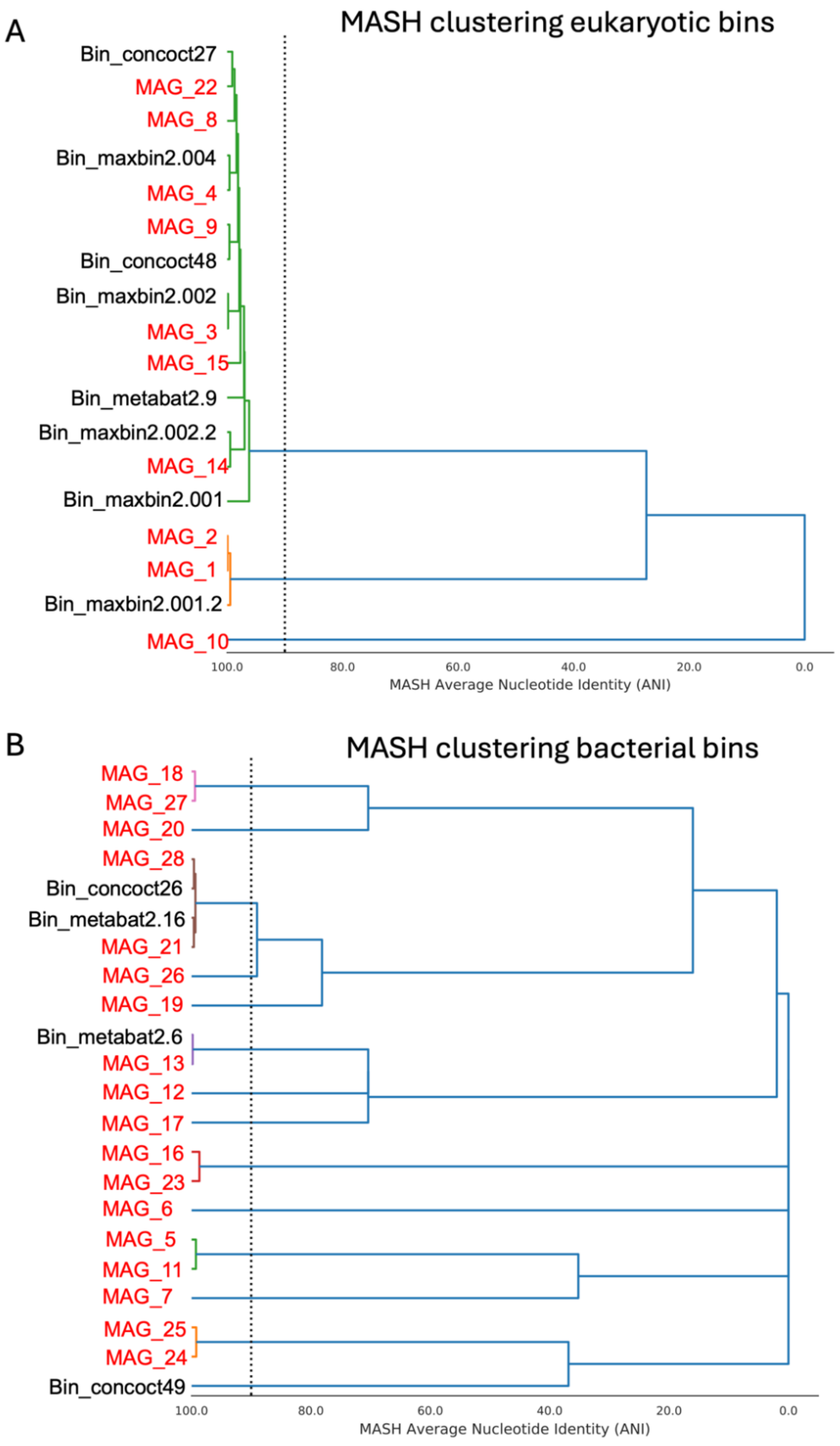


**Fig. S4. Metagenome-assembled metagenomes (MAGs) clustering.** De-replication of **A)** eukaryotic and **B)** bacterial bins with dRep results using two-steps algorithms, a rapid primary algorithm (MASH) and a more sensitive algorithm (ANI). Examples of genome relatedness figures generated by dRep. The black dotted line is the value of the lowest average nucleotide identity (ANI) in percentage resulting from a self-vs-self alignment of each genome in the cluster.

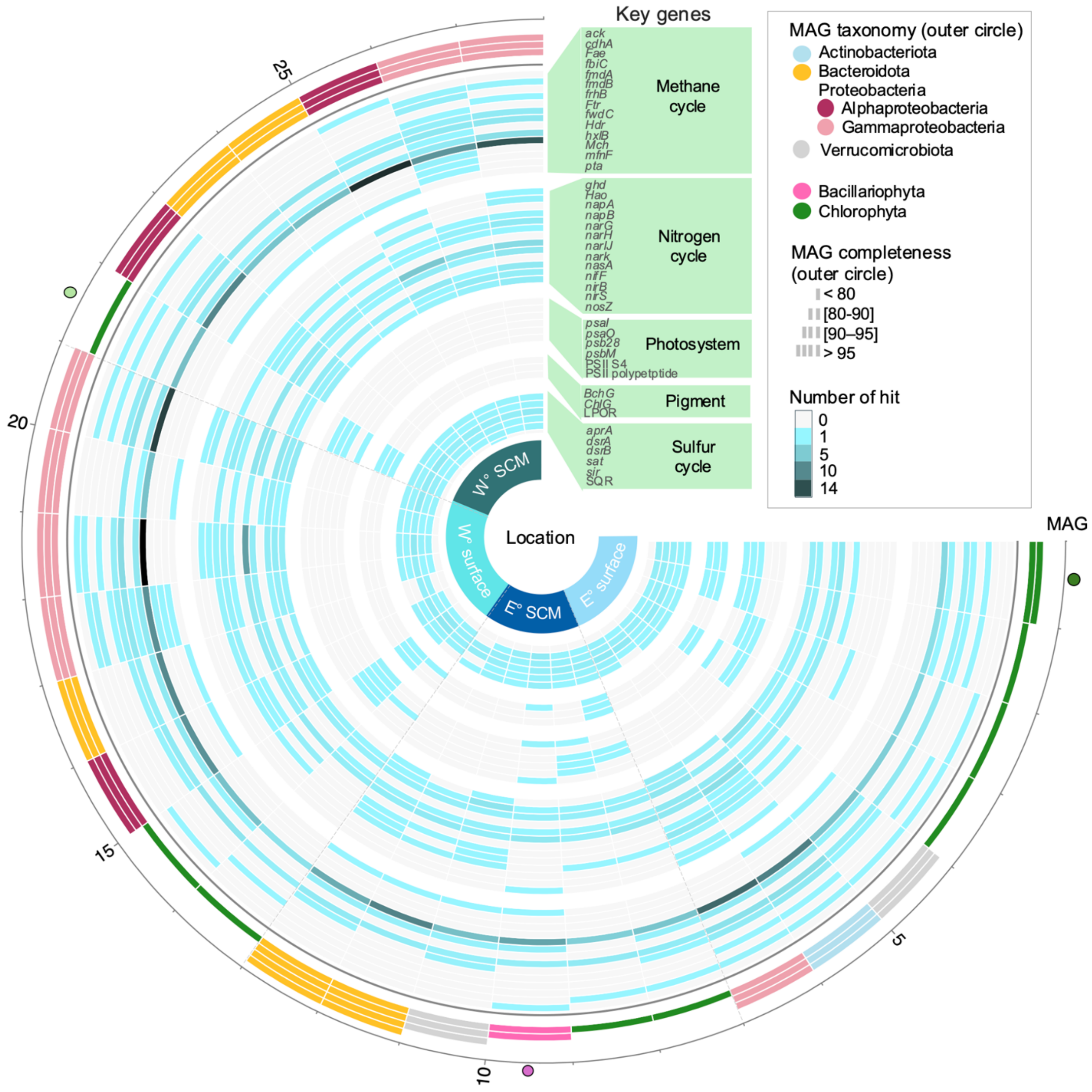


**Fig. S5. Methane, nitrogen and sulfur cycles genes with photosystem and pigment genes present in bacterial and eukaryotic metagenome-assembled genomes (MAGs).** Circular heatmap of selected key genes present in individual MAGs. Colors in the outer circle represent the taxonomic affiliations of each MAG (phylum and class levels). Colors in the inner circle represent the sampling site: either on the Canadian side of the North Water (W°) or on the Greenland side (E°). Depth of sampling is referred to as surface and subsurface chlorophyl maximum (SCM). The three selected MAGs (see **Fig. 7**) are indicated by a colored circle outside the outer circle of the circular heatmap. A corresponding table for each gene present in each MAG is in **Table S10**.

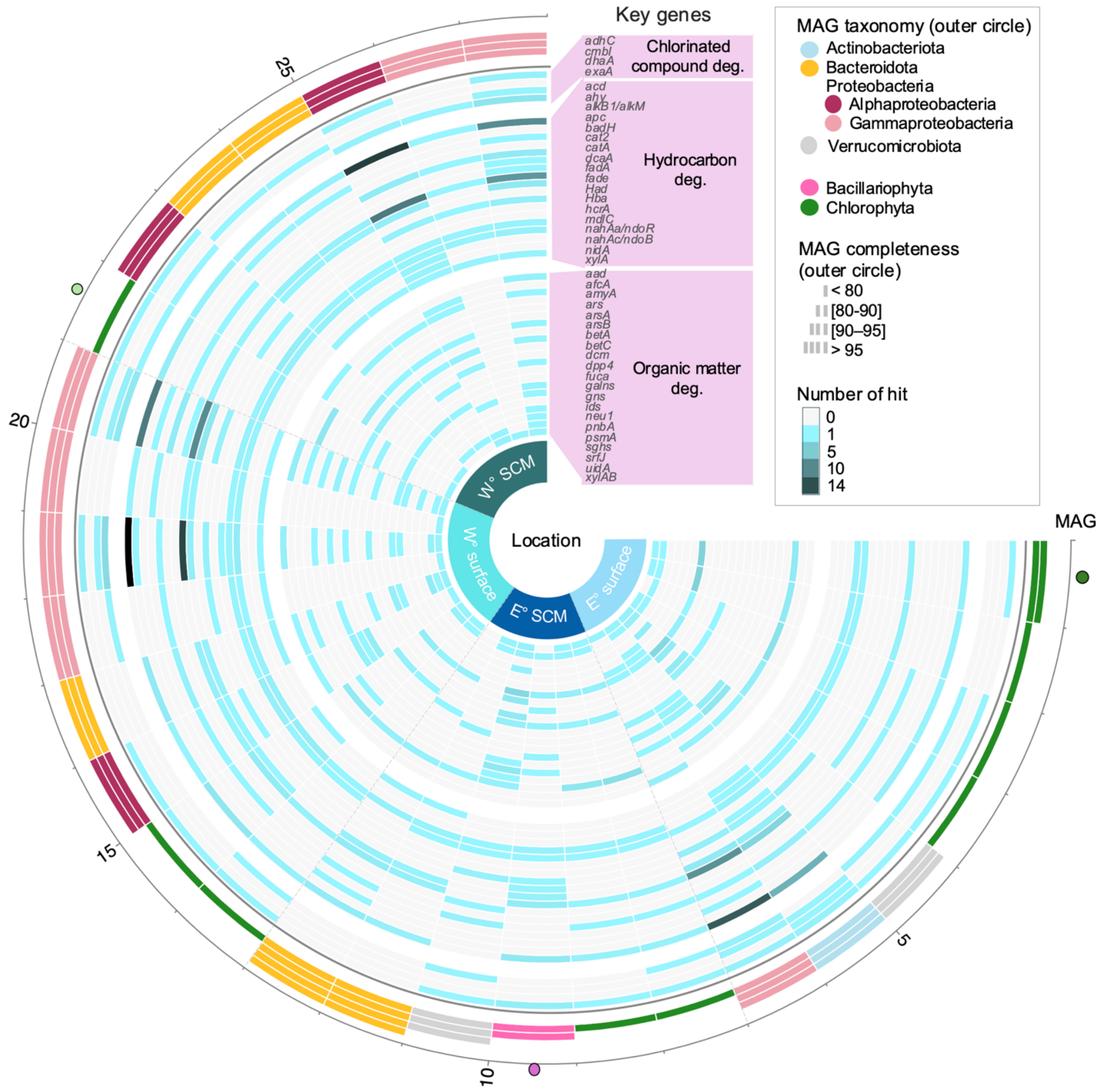


**Fig. S6. Hydrocarbon, chlorinated compound, and organic matter degradation genes present in bacterial and eukaryotic metagenome-assembled genomes (MAGs).** Circular heatmap of selected key genes present in individual MAGs. Colors in the outer circle represent the taxonomic affiliations of each MAG (phylum and class levels). Colors in the inner circle represent the sampling site: either on the Canadian side of the North Water (W°) or on the Greenland side (E°). Depth of sampling is referred to as surface and subsurface of chlorophyl maximum (SCM). The three selected MAGs (see **Fig. 7**) are indicated by a colored circle outside the outer circle of the circular heatmap. A corresponding table for each gene present in each MAG is in **Table S10**.

## REFERENCE


1. Freyria NJ. Aperçus taxonomiques et génomiques des communautés microbiennes face à un environnement fluctuant dans la Polynie des Eaux du Nord (Canada-Groenland): PhD thesis Laval University, Canada; (2021). https://doi.org/10.13140/RG.2.2.20405.55528.
2. Grasshoff K, Ehrhardt M, Kremling K. Methods of seawater analysis. 3rd. ref: Weinheim: Verlag Chemie GmbH; (1999).
3. Freyria NJ, Joli N, Lovejoy C. A decadal perspective on north water microbial eukaryotes as Arctic Ocean sentinels. Sci Rep. (2021) 11:1 p.8413. https://doi.org/10.1038/s41598-021-87906-4.
4. Marie D, Simon N, Guillou L, Partensky F, Vaulot D. DNA/RNA analysis of phytoplankton by flow cytometry. Curr Protoc Cytom. (2000) 11:1 p.11-2. https://doi.org/10.1002/0471142956.cy1112s11.
5. Marie D, Simon N, Vaulot D. Phytoplankton cell counting by flow cytometry. In: Andersen RA, editor. Algal culturing techniques. 1: Elsevier Academic Press; (2005). p. 253-67.
6. Belzile C, Brugel S, Nozais C, Gratton Y, Demers S. Variations of the abundance and nucleic acid content of heterotrophic bacteria in Beaufort Shelf waters during winter and spring. J Mar Syst. (2008) 74:3-4 p.946-56. https://doi.org/10.1016/j.jmarsys.2007.12.010.
7. Dasilva CR, Li WK, Lovejoy C. Phylogenetic diversity of eukaryotic marine microbial plankton on the Scotian Shelf Northwestern Atlantic Ocean. J Plankton Res. (2014) 36:2 p.344-63. https://doi.org/10.1093/plankt/fbt123.
8. Comeau AM, Li WK, Tremblay J-É, Carmack EC, Lovejoy C. Arctic Ocean microbial community structure before and after the 2007 record sea ice minimum. PloS One. (2011) 6:11 p.e27492. https://doi.org/10.1371/journal.pone.0027492.
9. Bushnell B. BBMap: A Fast, Accurate, Splice-Aware Aligner. Lawrence Berkeley National Lab.(LBNL), Berkeley, CA (United States); (2014). Contract No.: LBNL-7065E.
10. Rognes T, Flouri T, Nichols B, Quince C, Mahé F. VSEARCH: a versatile open source tool for metagenomics. PeerJ. (2016) 4 p.e2584. https://doi.org/10.7717/peerj.2584.
11. Edgar RC. Search and clustering orders of magnitude faster than BLAST. Bioinform. (2010) 26:19 p.2460-1. https://doi.org/10.1093/bioinformatics/btq461.
12. Schloss PD, Westcott SL, Ryabin T, Hall JR, Hartmann M, Hollister EB, et al. Introducing mothur: open-source, platform-independent, community-supported software for describing and comparing microbial communities. Appl Environ Microbiol. (2009) 75:23 p.7537-41. https://doi.org/10.1128/AEM.01541-09.
13. Quast C, Pruesse E, Yilmaz P, Gerken J, Schweer T, Yarza P, et al. The SILVA ribosomal RNA gene database project: improved data processing and web-based tools. Nucl Acids Res. (2012) 41:D1 p.D590-D6. https://doi.org/10.1093/nar/gks1219.
14. Guillou L, Bachar D, Audic S, Bass D, Berney C, Bittner L, et al. The Protist Ribosomal Reference database (PR2): a catalog of unicellular eukaryote small sub-unit rRNA sequences with curated taxonomy. Nucl Acids Res. (2012) 41:D1 p.D597-D604. https://doi.org/10.1093/nar/gks1160.

15. Alberti A. Data Descriptor: Viral to metazoan marine plankton nucleotide sequences from the Tara Oceans expedition. Sci Data. (2017) 4 p.170093. https://doi.org/10.1038/sdata.2017.93.
16. Bolger AM, Lohse M, Usadel B. Trimmomatic: a flexible trimmer for Illumina sequence data. Bioinform. (2014) 30:15 p.2114-20. doi: 10.1093/bioinformatics/btu170.
17. Caporaso JG, Kuczynski J, Stombaugh J, Bittinger K, Bushman FD, Costello EK, et al. QIIME allows analysis of high-throughput community sequencing data. Nat Methods. (2010) 7:5 p.335-6. https://doi.org/10.1038/nmeth.f.303.
18. Li D, Liu C-M, Luo R, Sadakane K, Lam T-W. MEGAHIT: an ultra-fast single-node solution for large and complex metagenomics assembly via succinct de Bruijn graph. Bioinform. (2015) 31:10 p.1674-6. https://doi.org/10.1093/bioinformatics/btv033.
19. Markowitz VM, Ivanova NN, Szeto E, Palaniappan K, Chu K, Dalevi D, et al. IMG/M: a data management and analysis system for metagenomes. Nucl Acids Res. (2007) 36 p.D534-D8. https://doi.org/10.1093/nar/gkm869.
20. West PT, Probst AJ, Grigoriev IV, Thomas BC, Banfield JF. Genome-reconstruction for eukaryotes from complex natural microbial communities. Genome Res. (2018) 28:4 p.569-80. https://doi.org/10.1101/gr.228429.117.
21. Kang DD, Li F, Kirton E, Thomas A, Egan R, An H, et al. MetaBAT 2: an adaptive binning algorithm for robust and efficient genome reconstruction from metagenome assemblies. PeerJ. (2019) 7 p.e7359. https://doi.org/10.7717/peerj.7359.
22. Wu Y-W, Tang Y-H, Tringe SG, Simmons BA, Singer SW. MaxBin: an automated binning method to recover individual genomes from metagenomes using an expectation-maximization algorithm. Microbiome. (2014) 2 p.1-18. https://doi.org/10.1186/2049-2618-2-26.
23. Alneberg J, Bjarnason BS, de Bruijn I, Schirmer M, Quick J, Ijaz UZ, et al. CONCOCT: clustering contigs on coverage and composition. arXiv. (2013) p.arXiv:1312.4038. https://doi.org/10.48550/arXiv.1312.4038.
24. Parks DH, Imelfort M, Skennerton CT, Hugenholtz P, Tyson GW. CheckM: assessing the quality of microbial genomes recovered from isolates, single cells, and metagenomes. Genome Res. (2015) 25:7 p.1043-55. https://doi.org/10.1101/gr.186072.114.
25. Saary P, Mitchell AL, Finn RD. Estimating the quality of eukaryotic genomes recovered from metagenomic analysis with EukCC. Genome Biol. (2020) 21 p.1-21. https://doi.org/10.1186/s13059-020-02155-4.
26. Olm MR, Brown CT, Brooks B, Banfield JF. dRep: a tool for fast and accurate genomic comparisons that enables improved genome recovery from metagenomes through de-replication. ISME J. (2017) 11:12 p.2864-8. https://doi.org/10.1038/ismej.2017.126.
27. Chaumeil P-A, Mussig AJ, Hugenholtz P, Parks DH. GTDB-Tk: a toolkit to classify genomes with the Genome Taxonomy Database. Bioinform. (2020) 36:6 p.1925–7. https://doi.org/10.1093/bioinformatics/btz848.
28. Dong X, Strous M. An integrated pipeline for annotation and visualization of metagenomic contigs. Front Genet. (2019) 10 p.999. https://doi.org/10.3389/fgene.2019.00999.
29. Package 'corrgram' [Internet]. 2018. Available from: https://github.com/kwstat/corrgram.
30. Package 'hmisc' [Internet]. 2019. Available from: https://hbiostat.org/R/Hmisc/.
31. Vegan: ecological diversity [Internet]. 2013. Available from: https://cran.r-project.org, https://github.com/vegandevs/vegan.
